# Room-temperature superprotonic conductivity in COOH-functionalized multicomponent covalent organic frameworks

**DOI:** 10.1039/d5sc06953j

**Published:** 2026-01-05

**Authors:** Gouri Chakraborty, Prasenjit Das, Biswajit Bhattacharya, Carsten Prinz, Franziska Emmerling, Arne Thomas

**Affiliations:** a BAM Federal Institute for Materials Research and Testing Richard-Willstätter-Str. 11 12489 Berlin Germany; b Department of Chemistry, Functional Materials, Technische Universität Berlin 10623 Berlin Germany arne.thomas@tu-berlin.de; c Department of Chemistry, Indian Institute of Technology Ropar Punjab 140001 India

## Abstract

In solid materials, the development of hydrogen bonding (H-bonding) networks within pores is crucial for efficient proton conductance. In this study, a chemically stable carboxylic acid-functionalized, quinoline-linked 2D microporous covalent organic framework (COF) (Qy-COOH) was synthesized using the Doebner multicomponent reaction (MCR) and compared to a similar framework lacking the –COOH functionality (Qy-H), prepared *via* an MC Domino reaction. The proton conductivity of the –COOH-functionalized MCR-COF was significantly enhanced, reaching 10^−2^ S cm^−1^, attributed to strong H-bonding interactions between water molecules and the dangling –COOH groups within the COF pores. In contrast, the analogous Qy-H framework exhibited a much lower proton conductivity of 10^−5^ S cm^−1^, while an imine-based COF showed only 10^−6^ S cm^−1^. This work represents the first demonstration of a general strategy to achieve efficient proton conduction in a class of layered 2D –COOH-functionalized COFs, offering superprotonic conductivity without requiring additives at room temperature. The MCR-COF design approach provides a promising pathway for developing highly stable and high-performance proton-conducting materials.

## Abstract 2

A –COOH-functionalized COF synthesized *via* the multicomponent Doebner reaction shows high proton conductivity from room to elevated temperatures. Dangling –COOH groups form H-bonded water networks, enabling Grotthuss proton hopping without dopants.

## Graphical abstract

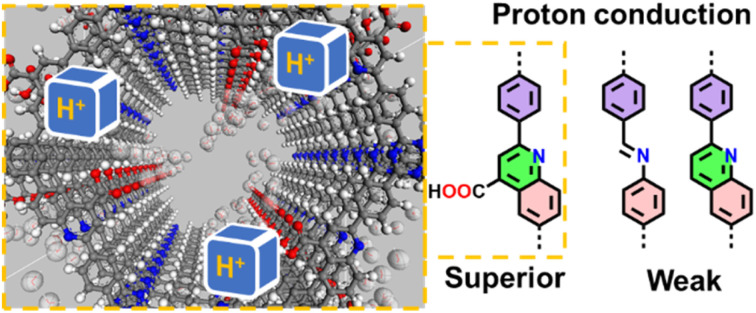

## Introduction

The growing global energy demand, driven by rapid industrialization and population growth, necessitates the development of greener, renewable energy systems to reduce the over exploitation of non-renewable natural resources such as fossil fuels. Fuel cells (FCs), which convert chemical energy into electrical energy with low to zero emissions, have garnered significant attention due to their high energy conversion efficiency, eco-friendly properties, fuel flexibility, and durability.^1,2^ Among various types of FCs, the proton exchange membrane fuel cell (PEMFC) stands out due to its superior performance.^3–6^ For this reason, developing proton-conducting materials is essential for membranes that play a key role in advanced technologies, especially in energy conversion and storage systems.^7,8^

Over the last 50 years, various polymers and polyelectrolytes have been explored as potential proton conductors to meet the growing demand for fuel cells and other electrochemical devices.^9–13^ However, these traditional proton-conductive materials suffer from their amorphous nature, are expensive to produce, and limited by narrow operational temperature ranges.^14,15^ This has led to new alternatives where long-range crystallinity, density, and flexibility in functionalization and operating temperatures are key factors in designing proton conductive materials. In particular, metal–organic frameworks (MOFs) have offered significant proton conductivities ranging from 10^−5^ to 10^−2^ S cm^−1^, *via* the incorporation of various proton carriers like counterions, acids, *N*-heterocycles, and other functional groups.^16^ However, MOFs often have limitations such as poor hydrolytic stability and low pH tolerance when doped with acidic entities during proton exchange in fuel cell membranes, limiting their practical application in proton-conductive systems.^17,18^

In recent years, covalent organic frameworks (COFs) – a novel category of porous crystalline polymers, in which organic building blocks are assembled into predetermined networks *via* covalent bonds,^19–21^ have been designed to achieve high proton conductivity with their ability to fine-tune nano spaces with different functionalities and offer flexible structure–activity relationships.^22^ An intriguing aspect is the propensity of 2D COFs to form uniform and well-defined 1D channels, similar to the water channels observed in Nafion, a perfluorinated sulfonated polymer widely regarded as industrial benchmark for its superprotonic conductivity (10^−1^ S cm^−1^) at 60–80 °C under high 98% relative humidity (RH).^23^ Several strategies for COF-based proton conductors involve either: (a) doping with external proton carriers such as mineral acids (*e.g.*, H_3_PO_4_, H_2_SO_4_),^24–26^ organic acids (*e.g.*, phytic acid, *p*-toluene sulfonic acid (PTSA)),^27,28^ polyoxometalates (PW_12_)^29^ and *N*-heterocycles (*e.g.*, imidazole, triazole),^30^ or (b) covalently integrating acidic groups (*e.g.*, sulfonic or hydroxyl groups)^31–34^ directly into the framework. However, the doping approach poses difficulties in controlling proton carrier loading resulting in weak host–guest interaction, while the covalent attachment of acidic groups within COF structures remains synthetically demanding. Additionally, till now only –SO_3_H and –OH acidic functionalities have been used to develop proton conducting COFs which creates a scope to explore other acidic functional groups.^31–34^ Proton-conducting COFs have been developed by post-synthetic modification (PSM) using the two strategies mentioned, which however often lead to non-uniform dopant or functional group distribution causing leaching issues and inconsistent results.^22^ However, only a limited number of studies have employed carboxylic acid (–COOH) groups as proton carriers to construct intrinsically proton-conductive COFs, using PSM under harsh hydrolysis conditions.^35^ Moreover, these approaches are only feasible for a few highly robust COFs, as many frameworks are prone to decomposition under the severe conditions required for hydrolysis. In this context, the direct construction of COOH-functionalized COFs through a pre-assembly strategy is highly desirable, as it avoids the drawbacks associated with post-modification while enabling a uniform and dense distribution of proton-conducting sites. Furthermore, employing building blocks rich in –COOH groups provide following advantages: (i) ensure abundant intrinsic proton sources and a continuous hydrogen-bonding (H-bonding) network; (ii) create an extended array of proton conduction pathways; and (iii) form long-range ordered one-dimensional (1D) channels that promote rapid proton migration.^36^ Additionally, COFs with high proton conductivity under anhydrous environment are known, but these require high temperature and materials need to sustain harsh environments.^30,37–41^ In contrast, humid or wet environments provide high conductivity as water facilitates excellent proton transport with reduced resistance and consequently such materials are widely used in low temperature PEMFCs for vehicles, portable electronics and stationary power supply, as well as humidity-sensitive sensors. However, so far an optimal combination of high conductivity, well-defined conduction pathways, stability, and suitable operating conditions is very challenging to achieve, highlighting a clear demand for new advancements in proton-conducting COF materials *via* novel synthesis approaches. COF-based membranes promise to overcome the limitations of traditional polymer proton conductors by offering crystalline, well-ordered channels that enable fast and directional proton transport. Their superior thermal and chemical stability also allows operation over a wider range of temperatures and humidity than polymer-based systems. Although COF synthesis may seem costly at the laboratory scale, their modular design and scalable fabrication routes – using inexpensive linkers and simple synthetic methods – can significantly reduce costs for large-scale production.

To date, the molecular design of proton-conducting COFs has mostly focused on Schiff base condensation, which requires the use of β-ketoenamine linkages to stabilize imine bonds against harsh chemical conditions.^42^ Recently, novel, highly stable COFs have been developed using a fascinating linkage chemistry: multi-component reactions (MCRs).^43–50^ This approach is emerging as an atom economic strategy, allowing a straightforward synthetic route by fusing multiple monomers as building blocks in a single process to create robust aromatic linkages, giving COFs remarkable chemical and thermal stability with tailorable pore functionalities.^51–54^ Unlike other synthesis methods used in COFs, MCRs allow a defined orientation of functional groups along the pore channels making them available for interaction with guest molecules entering the pores. However, till date this synthetic method has not been utilized for generating proton conductive COFs. We therefore envisioned to use a MCR for the formation of COFs equipped with proton carriers along the pores. In the present work, quinoline-linked COFs with carboxylic acid (–COOH) groups pointing into the pore channels are synthesized using the Doebner MCR. These COFs show high proton conductivity (∼10^−3^ to 10^−2^ S cm^−1^) across a wide range of operating temperatures under humid conditions. Furthermore, analogous COFs without –COOH functionality, namely quinoline- and imine-linked COFs, prepared *via* Domino MCR and Schiff base imine reaction, have been synthesized and their proton conductivities are compared to verify the significant role of –COOH group in enhancing proton conduction. The –COOH-functionalized MCR-COFs exhibit the highest proton conductivity among COF-based materials under hydrous conditions, without the need for any additives.

## Results and discussion

**Fig. 1:**
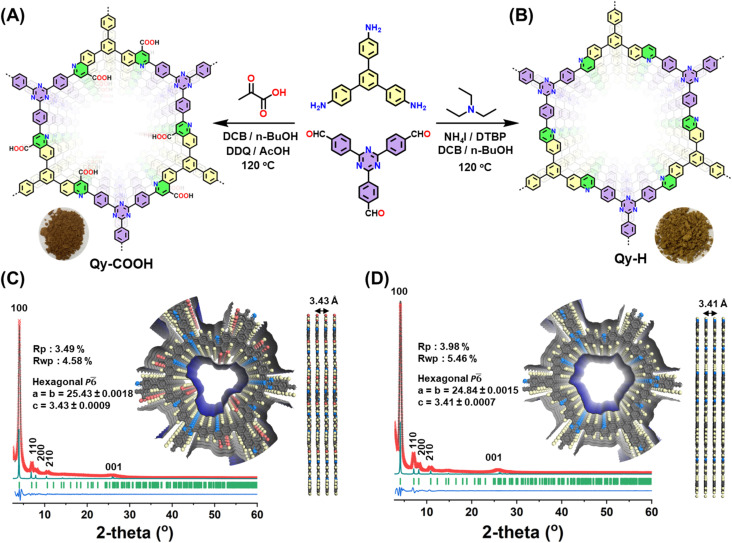
Synthesis, structure, and characterization of Qy-COOH and Qy-H: synthesis scheme for (A) Qy-COOH and (B) Qy-H; PXRD patterns for (C) Qy-COOH and (D) Qy-H: experimental (black dotted line) and Pawley refined (red cross) patterns, Bragg position (green) and corresponding difference plot (blue line). The theoretical PXRD pattern for an eclipsed AA stacking model of Qy-COOH and Qy-H is provided as cyan line. Top and side view of AA stacking in Qy-COOH and Qy-H. (Color code: C, gray spheres; O, red spheres; N, blue spheres, H, pale yellow spheres).

To understand the impact of pore functionality in the MCR-COFs, two comparable COFs, one with the pure quinoline linkage and the other with quinoline linkages and COOH-groups pointing into the pores were synthesized *via* the three-component one-pot Domino and Doebner reaction, respectively. Both reactions involve trivalent aldehyde- and amine-functionalized monomers, while as third component triethylamine (Domino) or pyruvic acid (Doebner) is used (Fig. 1A). To verify the successful one-pot Domino three component [4 + 2] cyclic condensation reaction, we synthesized a model compound using 4-methoxy aniline, 4-fluorobenzaldehyde and triethylamine and characterized it using a variety of techniques (Scheme S1 and Fig. S1, S2). Qy-COOH was synthesized *via* the three-component Doebner reaction, using 2,4,6-Tris(4-aminophenyl)benzene (TAB), 4,4′,4″-(1,3,5-triazine-2,4,6-triyl)tribenzaldehyde (TTA) and pyruvic acid in a solvent mixture of *o*-dichlorobenzene/*n*-butanol (1 : 1), and cat. 2,3-dichloro-5,6-dicyano-1,4-benzoquinone (DDQ) at 120 °C for 72 h. The 4-COOH-quinoline-linked COF was collected as a greenish brown-colored powder in 86% yield (Fig. 1 and Scheme S2). On the other hand, Qy-H was synthesized using TAB, TTA and triethylamine and after optimization of various parameters (*e.g.* solvents, catalyst, and reaction temperature), using a solvent mixture of *o*-dichlorobenzene/*n*-butanol (1 : 1), and ammonium iodide (NH_4_I), di-*tert*-butyl peroxide (DTBP) and cat. 2,3-dichloro-5,6-dicyano-1,4- benzoquinone (DDQ) at 120 °C for 72 h, we achieved corresponding highly crystalline olive-colored COFs with high yield and crystallinity (93%) (Fig. 1 and Scheme S3). To study the effect of the quinoline and the –COOH functionality for proton conductance, an analogous imine COF (Im-1, yellow color) was prepared as well (Scheme S4).

Using powder X-ray diffraction (PXRD) analysis, the crystallinity of both Qy-COOH and Qy-H was measured and confirmed (Fig. 1). The experimental diffraction pattern displayed reflections consistent with those expected for a 2D layered structure (Fig. 1C). A characteristic reflection in the low-angle region of 2*θ* at 4.1° with high intensity was observed for both COFs (Fig. 1C). In addition, a series of lower symmetry reflections was obtained in the PXRD patterns which were not ascribable to the starting monomers, implying the successful generation of a crystalline framework. To investigate the structural arrangement, hexagonal layered models with a honeycomb (hcb) topology based on different stacking sequences were constructed and optimized geometrically. The comparison of experimental and theoretical PXRD suggests that the studied frameworks adopt a 2D hexagonal structure with an eclipsed AA stacking pattern along the *c*-axis. Pawley refinement across the full diffraction profile was conducted to refine the unit cell parameters, supporting the proposed structural model (Tables S2 and S3).

**Fig. 2:**
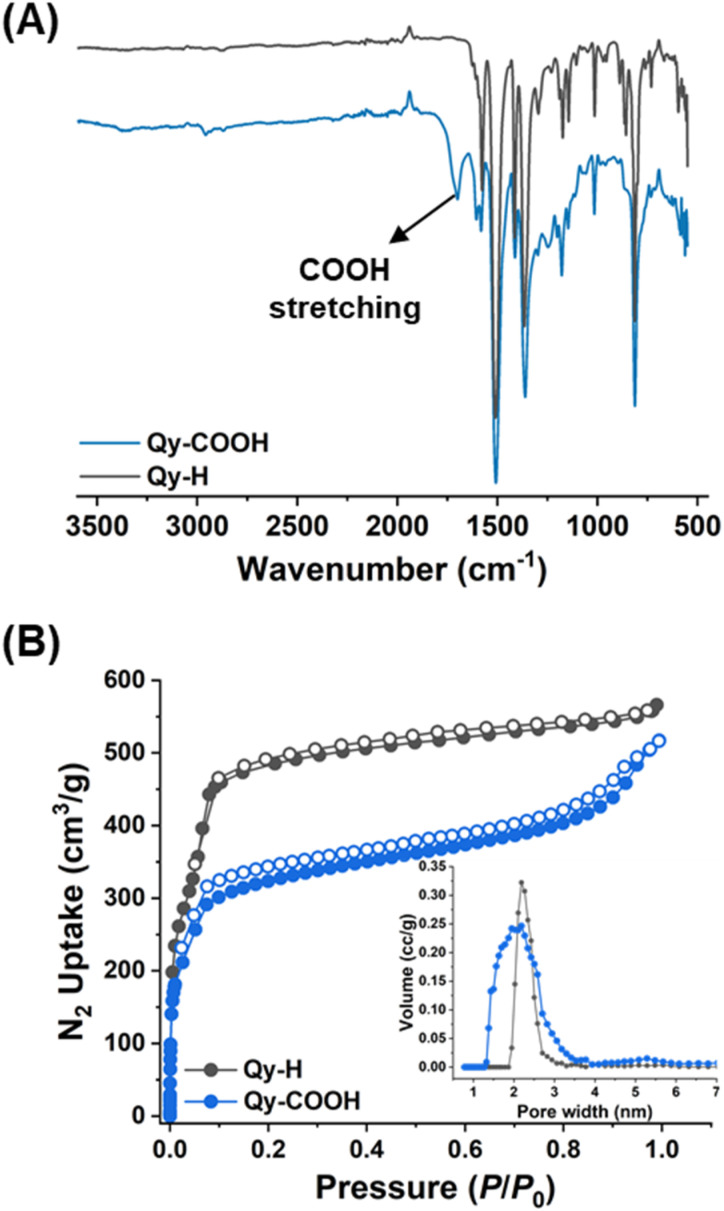
(A) FTIR spectra for Qy-COOH and Qy-H; (B) N_2_ sorption at 77 K for Qy-COOH and Qy-H; (inset) pore size distribution of Qy-COOH and Qy-H.

Both COFs were fully characterized by numerous analytical techniques such as Fourier transform infrared (FTIR), X-ray photoelectron spectroscopy (XPS), solid-state cross-polarization magic-angle-spinning (CP-MAS) ^13^C-NMR, thermogravimetric analysis (TGA) and porosity and surface area analysis. The CP-MAS ^13^C-NMR spectra of Qy-COOH and Qy-H revealed signals characteristic of quinoline groups, appearing at 155 and 152 ppm, respectively (Fig. S3). Additionally, broader signals at 170–167 ppm were observed for Qy-COOH compared to Qy-H, attributed to the presence of both –COOH and triazine groups in Qy-COOH, whereas Qy-H contains only triazine. In both cases, the peak corresponding to imine (157 ppm) was absent, indicating the successful progression of the multicomponent reaction. The FTIR spectrum shows characteristic peaks at 1579 and 1574 cm^−1^, attributed to the C=N stretching frequencies from the quinoline rings of Qy-COOH and Qy-H, respectively (Fig. 2A).^48^ Additionally, Qy-COOH exhibits a distinct peak at 1720 cm^−1^, corresponding to the C=O stretching frequency of the –COOH group, which is not present in either Qy-H or Im-1 (Fig. S4). The formation of quinoline linkages was further validated by XPS, where the N 1s spectra showed a characteristic peak at 400 eV for both COFs, consistent with previous reports (Fig. S5 and S6).^48,49^ Additionally, the O 1s XPS spectrum of Qy-COOH displayed an extra C=O peak at 534 eV, which was absent in Qy-H, showing the presence of the –COOH group (Fig. S5 and S6).

TGA revealed no significant weight loss for Qy-H up to 500 °C under a N_2_ atmosphere, whereas Qy-COOH exhibited a gradual weight decrease beginning at 200 °C, attributed to the decomposition of the –COOH groups (Fig. S7). However, both COFs demonstrated superior chemical stability compared to their imine-based analogue. Stability tests showed that both COFs remained intact after exposure to 6 M HCl and 6 M NaOH for 7 days, as confirmed by PXRD analysis (Fig. S8).

**Fig. 3:**
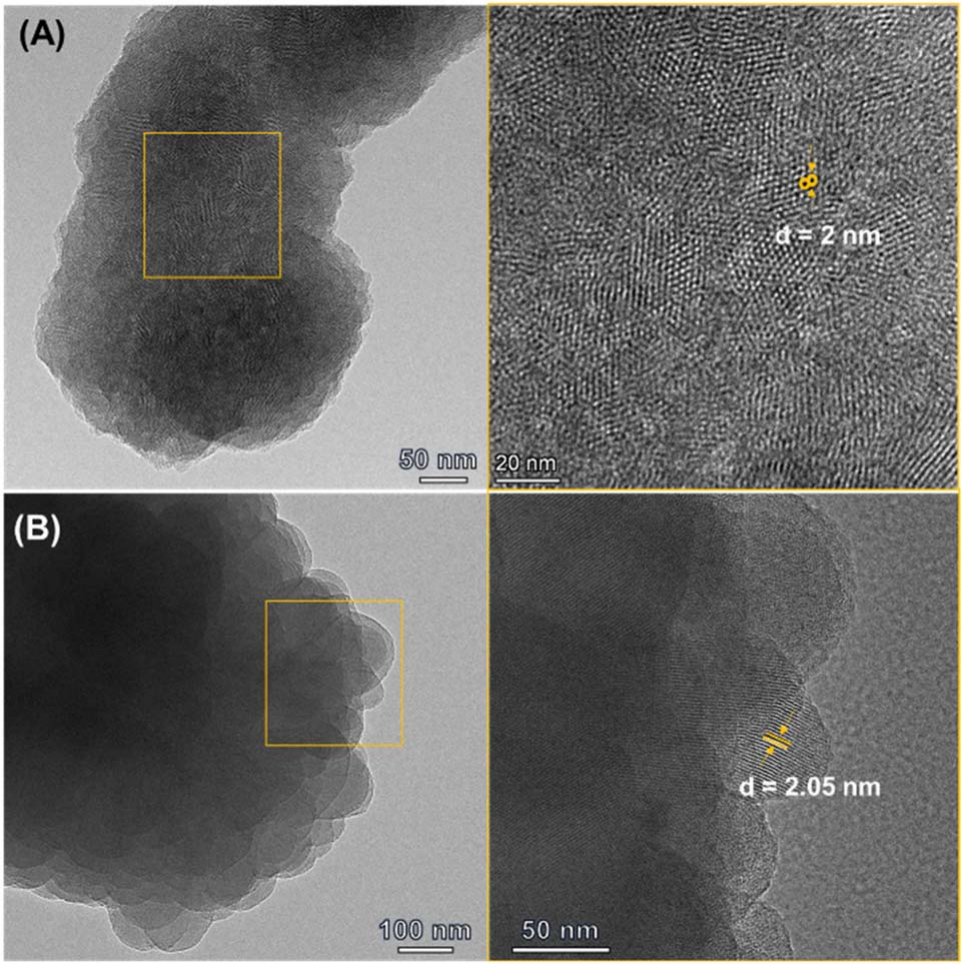
(A) HRTEM image of Qy-COOH; (B) HRTEM image of Qy-H.

Nitrogen sorption measurements were conducted at 77 K to evaluate the porosity of Qy-COOH and Qy-H, revealing typical type I reversible sorption isotherms (Fig. 3C). The Brunauer–Emmett–Teller (BET) surface area and nitrogen uptake were lower for Qy-COOH compared to Qy-H and even lower than Im-1, further highlighting the structure–property relationship. For Qy-COOH, the BET surface area was measured to be 897 m^2^ g^−1^, compared to 1666 m^2^ g^−1^ for Qy-H and 1900 m^2^ g^−1^ for Im-1 (Fig. S9). This reduction can be partially explained by the decreased molecular weight of the repeating units within this series. Furthermore, the presence of pendant carboxylic acid groups, which are pointing into the pores reduce gravimetric nitrogen uptake. This partial pore filling can be also seen in the pore sizes of the COFs, calculated using density functional theory (DFT), were a broad range from 1.40 to 2 nm is obtained for Qy-COOH, while for Qy-H a narrow PSD at 2 nm is observed (Fig. 2B, inset). These values align well with the pore sizes predicted from the structural models, supporting the experimental observations.

To examine the morphology and pore structure, field emission scanning electron microscopy (FESEM) and high-resolution transmission electron microscopy (HRTEM) analyses were performed for both COFs. The FESEM images revealed that Qy-COOH exhibits a uniform assembly of crystalline nanorods forming microflowers, whereas Qy-H shows a rather undefined microstructure (Fig. S10 and S11). HRTEM analysis of both COFs confirmed the periodic hexagonal structure and lattice fringes (Fig. 3). These results were consistent with the simulated PXRD patterns.

**Fig. 4:**
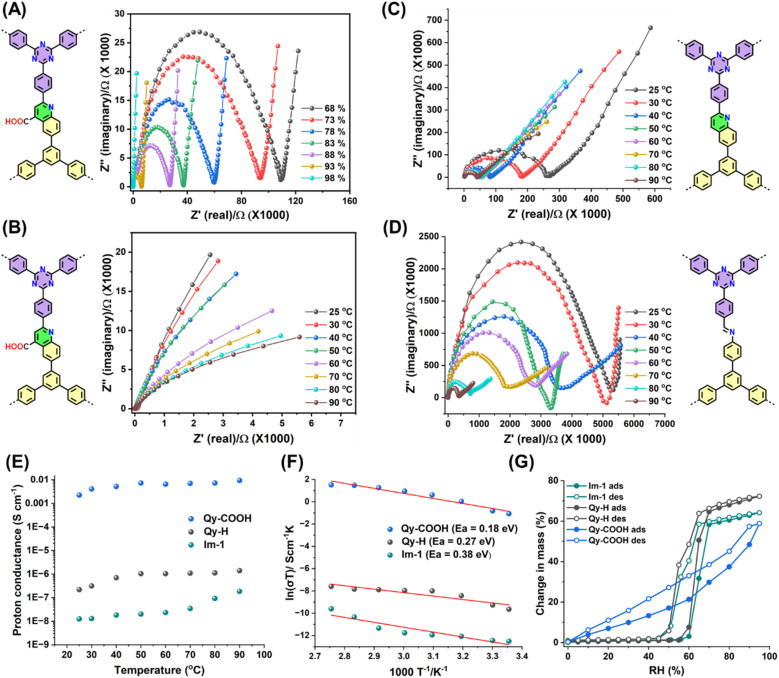
(A) Nyquist plots of Qy-COOH under different RH at RT. Nyquist plots of (B) Qy-COOH, (C) Qy-H and (D) Im-1 measured under 98% RH at different temperatures showing the temperature dependent proton conductivities. (E) Proton conductivity of all COFs at different temperature under 98% RH. (F) Arrhenius plots for Qy-COOH, Qy-H and Im-1 at different temperatures. (Dots for data and curves for fitting). (G) Water sorption isotherms of Qy-COOH, Qy-H and Im-1 at 298 K.

The presence of well-defined, COOH-functionalized pores within an open framework structure with high chemical stability prompted us to investigate the proton conductivity of these materials. Proton conductivity measurements of Qy-COOH, Qy-H and Im-1 were performed using alternating current electrochemical impedance spectroscopy (EIS). First pellets were pressed with uniform diameter (5.0 mm) and thickness (0.5 mm) and were subjected to humidification in a chamber at different temperatures.^55^ Impedance spectra for proton conductivities of all COFs were measured under 68–98% RH at 25 °C as well as temperatures ranging from 25 to 90 °C under 98% RH (Fig. 4). The Nyquist plots exhibit an incomplete semicircle at high frequencies, indicative of dominant ionic conductivity and bulk resistance within the material. The presence of a low-frequency tail further suggests proton accumulation at the electrode interface, consistent with blocking behavior.^56^

Nyquist plots show that Qy-COOH exhibits low proton conductivity in the range of 10^−7^–10^−6^ S cm^−1^ under low RH at room temperature. As RH increases, resistivity decreases. At 98% RH, Qy-COOH shows superprotonic conductivity, achieving a value of 1.14 × 10^−3^ S cm^−1^ (Fig. 4A). A similar trend is observed with rising temperature (25 to 90 °C) under constant 98% RH, where proton conductivity increases as expected. At 90 °C and 98% RH, Qy-COOH reaches a proton conductivity of 9.25 × 10^−3^ S cm^−1^ in its pristine form (Fig. 4B). This is among the highest values reported for COF-based materials without dopants and is comparable to polymer-based proton conductors (Table S1). The analogous COFs, Qy-H and Im-1, also show increased conductivity with rising RH and temperature, but their absolute values are significantly lower (Fig. 4C–E). Specifically, the proton conductivity follows the trend: Qy-COOH (9.3 × 10^−3^ S cm^−1^) > Qy-H (10^−7^ S cm^−1^) > Im-1 (10^−8^ S cm^−1^), that is, Qy-COOH is about 10^4^ or 10^5^ times more conductive than Qy-H and Im-1. These results highlight the importance of the COOH-groups, which likely engage in strong hydrogen bonding with water molecules, facilitating proton transport. Water contact angle measurements support this interpretation. Due to its highly hydrophilic nature, for Qy-COOH no contact angle can be measured as the water droplet is directly soaked into the material, while Qy-H and Im-1 exhibited contact angles of 23° and 43°, respectively (Fig. S12).

As mentioned, the proton conduction is temperature dependent and with increased temperature proton conductivity increases. Therefore, proton conductivities follow the Arrhenius equation of *σ*(*T*) = *σ*^0^ e^−*E*_a_/*RT*^, where *σ*(*T*) is the proton conductivity at a specific temperature *T*, *σ*^0^ is the pre-exponential factor, *E*_a_ is the activation energy (eV), *R* is the universal gas constant (8.3144 J mol^−1^ K^−1^), and *T* is the absolute temperature (K). Plotting log *σ versus* inverse temperature (*T*^−1^) yields linear curves for all COFs (Fig. 4F). From their slopes, the *E*_a_ was calculated to be 0.18, 0.27, and 0.38 eV for Qy-COOH, Qy-H and Im-1, respectively. The *E*_a_ values of Qy-COOH and Qy-H indicate a Grotthuss mechanism, where proton conduction is predominated by a hopping mechanism due to the presence of –COOH and quinoline groups allowing strong H-bonding.

The higher H-bonding interactions in the COFs enhance proton conductivity under relative humidity (RH) conditions and facilitate proton hopping within the pores.^57^ In case of Im-1, the calculated values suggest a dominant Grotthuss-type proton conduction, possibly with some contribution from a vehicle-type mechanism. To support this hypothesis and validate the obtained results, water sorption measurements were performed. Qy-H and Im-1 exhibited typical type IV sorption isotherms with minimal hysteresis, with water uptake starting at *p*/*p*_0_ = 0.6 (Fig. 4G), correlating with their proton conductivity trends. In contrast, Qy-COOH displayed a reduced overall water uptake but starting at lower pressures, accompanied by a significant hysteresis (Fig. 4G). This behaviour proves stronger interactions between the water molecules within the pores, promoting better proton conductivity. The overall water uptake with the total amounts of adsorbed water molecules at *p*/*p*_0_ ∼ 0.9 is 70, 77, and 63% change in mass for Im-1, Qy-H, and Qy-COOH, respectively. The enhanced interaction between water and the carboxylic acid groups in Qy-COOH was further verified by theoretical calculations.

**Fig. 5:**
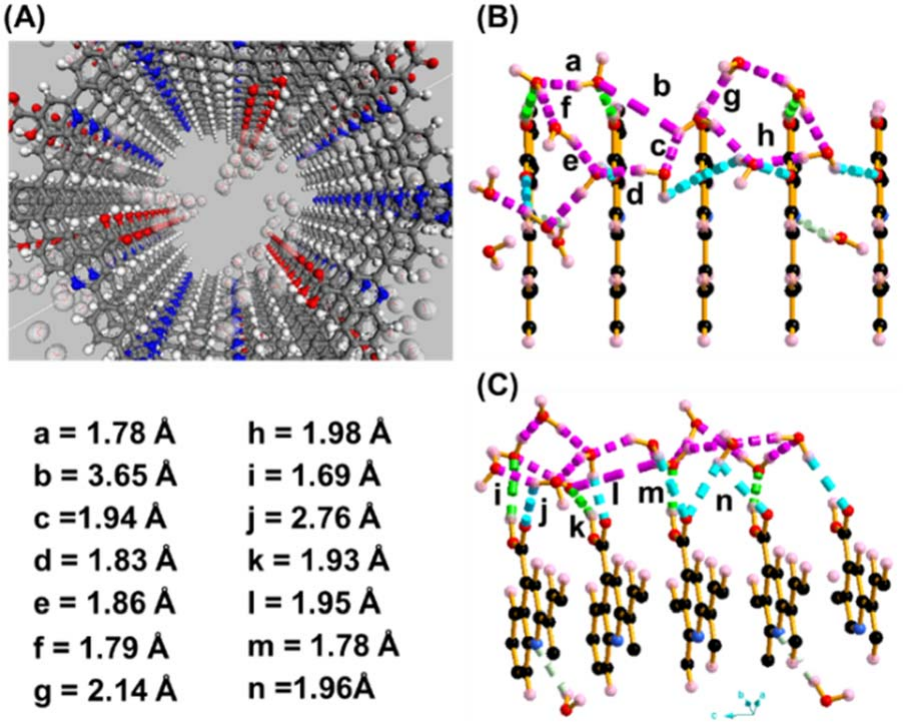
CBMC simulation to understand the interaction of water with Qy-COOH: (A) formation of water channel in *c*-axis through extensive H-bonding with water molecule. (B and C) Top and sidewise view of extended H-bonded tunnels with –COOH group.

To gain atomistic insights into these phenomena, configurational-bias Monte Carlo (CBMC) simulations were performed at 298 K and 0.1 bar using the Materials Studio suite. The simulations revealed that Qy-COOH exhibited a higher water uptake at lower pressure than both Qy-H and Im-1, validating the experimental observations (Fig. S13–S15). The synergistic role of the carboxylic acid moiety and the quinoline nitrogen atom facilitates the formation of extensive hydrogen-bonding networks. The C=O and O–H groups of the –COOH unit act as dual hydrogen bond acceptor and donor sites, respectively, enabling the formation of a zigzag hydrogen-bonded motif spanning multiple layers along the crystallographic *c*-axis (Fig. 5 and S13). Both the C=O and O–H groups of the –COOH moiety simultaneously formed hydrogen bonds with two guest water molecules (C=O⋯OW = 1.6–1.8 Å) and (O–H⋯OW = 1.9–2.5 Å). These guest water molecules further engage in hydrogen bonding with adjacent layers, assembling into a well-defined pseudo-oxacyclohexane-like six-membered H-bonded ring, which bridges three neighboring COF layers. Furthermore, guest water molecules interact with the electron-rich nitrogen atoms of the quinoline rings of Qy-COOH (N⋯OW = 2.65 Å) and Qy-H (N⋯OW = 2.78 Å), reinforcing both intra- and interlayer hydrogen bonding. This synergistic interaction is significantly more pronounced in Qy-COOH than in Qy-H, highlighting the critical role of the –COOH group in enhancing structural water retention and facilitating long-range proton conduction *via* the Grotthuss mechanism. In contrast, Im-1, possessing only imine nitrogen sites, displays relatively weaker H-bonding interactions (N⋯OW = 2.84 Å), resulting in less efficient proton transport pathways. Taken together, these results underline the importance of functionalization of the MCR-COFs for increased water affinity and proton conductivity.

In addition to the pore environment, crystallinity and surface area also plays an important role.^58^ For example, when Qy-COOH is attempted to be prepared from Im-1*via* PSM (see SI), both crystallinity and surface area decrease. Furthermore, PSM of all imine linkages is not complete. As a result, proton conductivity in this material is lower (10^−6^ S cm^−1^), highlighting the importance of the direct multicomponent reaction to prepare the proton conducting COFs.

To elucidate the general role of the –COOH functional group in enhancing proton conductivity, we synthesized an additional Doebner COF by modifying the aldehyde precursor to nitrogen-containing terphenyl core (4,4′,4″-(amino-2,4,6-triyl)tribenzaldehyde) (Qy-COOH1; Scheme S5 and Fig. S19–S22, Table S4). Notably, Qy-COOH1 exhibited an even slightly increased proton conductivity at room temperature (∼2.2 × 10^−3^ S cm^−1^) and at elevated temperatures under 98% RH (∼1.25 × 10^−2^ S cm^−1^) compared to Qy-COOH, with an activation energy (*E*_a_) of 0.08 eV, consistent with a Grotthuss-type conduction mechanism (Fig. S23).

**Fig. 6:**
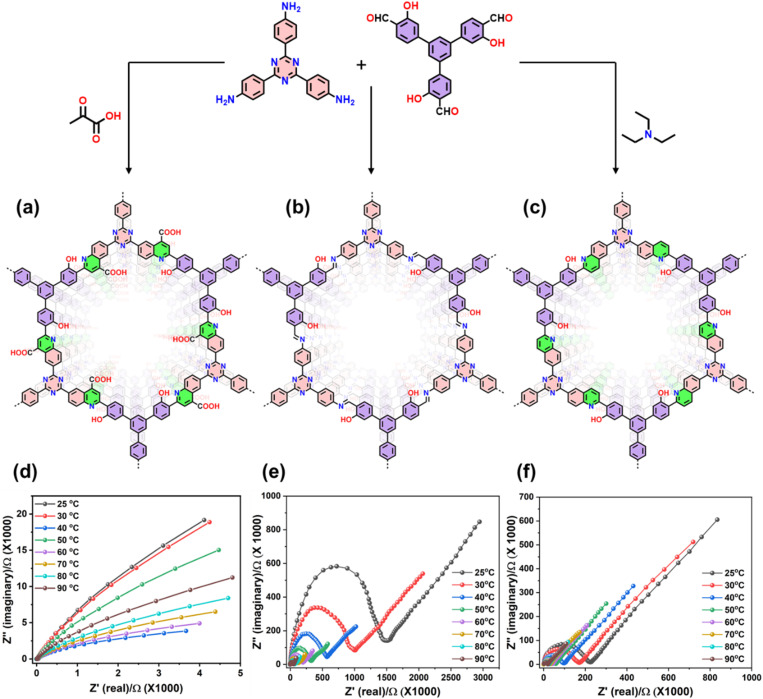
Synthesis and structure of (a) OH-Qy-COOH, (b) OH-Im and (c) OH-Qy-H. Nyquist plots of (d) OH-Qy-COOH, (e) OH-Qy-H and (f) OH-Im measured under 98% RH at different temperatures showing the temperature dependence of proton conductivities.

Furthermore, we investigated the influence of additional hydrophilic and hydrogen bonding functional groups, namely hydroxy groups. The incorporation of hydroxy functionality *via* the aldehyde precursor 5′-(4-formyl-3-hydroxyphenyl)-3,3″-dihydroxy-[1,1′ : 3′,1″-terphenyl]-4,4″-dicarbaldehyde yielded a series of analogous COFs: OH-Im (imine-linked), OH-Qy-H (quinoline-based), and OH-Qy-COOH (quinoline-4-carboxylic acid-based). The synthetic details and full characterization are provided in the SI (Fig. 6, Schemes S6–S8, Fig. S24–S31 and Tables S5–S7). The proton conductivity values followed the same trend as their non-hydroxy analogs, again with slight enhancements: OH-Im (10^−7^ S cm^−1^), OH-Qy-H (10^−6^ S cm^−1^), and OH-Qy-COOH (2.9 × 10^−3^ S cm^−1^) at room temperature and 98% RH (Fig. 6 and S32). At 90 °C and 98% RH, OH-Qy-COOH exhibited a proton conductivity of 1.3 × 10^−2^ S cm^−1^, showing further improvement over Qy-COOH (Fig. 6). Water sorption studies and activation energy analyses followed similar trends, with calculated *E*_a_ values of 0.10, 0.37, and 0.50 eV for OH-Qy-COOH, OH-Qy-H, and OH-Im, respectively (Fig. S32 and S33). The *E*_a_ values for OH-Qy-COOH confirm a Grotthuss-type proton hopping mechanism. In contrast, OH-Qy-H exhibits a mixed mechanism involving dominant Grotthuss and with some vehicle-type proton transport, while OH-Im primarily follows a vehicle-type mechanism. These observations highlight the crucial role of the –COOH group in promoting efficient proton transfer within the pore environment. Overall, these COFs exhibit the second-highest intrinsic proton conductivity among reported proton-conducting COFs and polymers, next only to the imidazole-based COF (BIP) (Fig. S35 and Table S1).

**Fig. 7:**
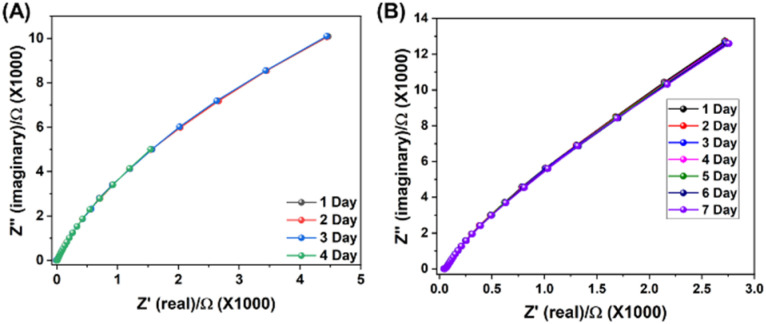
Time dependent Nyquist plots of (A) Qy-COOH measured at 80 °C and under 95% RH; (B) OH-Qy-COOH measured at 30 °C and under 95% RH.

In addition, the long-term stability and recyclability of these MCR-COFs were also evaluated. Time-dependent proton conductivity measurements of Qy-COOH and OH-Qy-COOH over a period of 4 and 7 days revealed negligible degradation, indicating excellent operational stability (Fig. 7). Moreover, post-cycling PXRD, FTIR and XPS analyses confirmed that the materials retained their crystallinity and structural integrity, establishing MCR-COFs as highly robust platforms for proton conduction applications (Fig. S34). However, in case of imine COF the crystallinity was almost lost after first cycle of all experiments (Fig. S34). This work therefore presents the first example of pristine 2D MCR-COFs exhibiting high proton conductivity, reaching values comparable to or exceeding many of the best-known proton-conducting porous materials reported to date.

## Conclusion

In conclusion, –COOH-functionalized COFs, prepared by the multicomponent Doebner reaction, exhibit a superprotonic conductivity at RT. The comparison with analogous quinoline or imine COFs without carboxylic groups show that –COOH functionalization enhances proton conductivity promoted by strong H-bonding with water molecules in the pore channels. The high chemical stability of these MCR-COFs ensures high and durable proton conductivity, meeting practical requirements. These findings highlight the critical role of pore functionality, high crystallinity and surface area in optimizing proton conductivity in COFs.

## Author contributions

P. D. and A. T. in collaboration with G. C. and B. B. conceived the project. P. D. and G. C. designed the experiments. P. D. and G. C. performed the synthesis and characterization of the COFs. B. B., P. D. and G. C. carried out the proton conductance measurement. C. P. and P. D. performed the TEM and water sorption. G. C, P. D. and A. T. wrote the manuscript. A. T. and F. E. supervised the project. All authors contributed to the discussions and gave comments on the manuscript.

## Conflicts of interest

There are no conflicts to declare.

## Supplementary Material

SC-OLF-D5SC06953J-s001

## Data Availability

The data supporting this article have been included as part of the supplementary information (SI). Supplementary information is available. See DOI: https://doi.org/10.1039/d5sc06953j.
