# Supplementary material for: Room-temperature superprotonic conductivity in COOH-functionalized multicomponent covalent organic frameworks
Source: Chem Sci. 2026 Jan 5;17(8):4107–15. doi: 10.1039/d5sc06953j (PMC12766738; doi:10.1039/d5sc06953j)
Supplement: SC-017-D5SC06953J-s001 [file SC-017-D5SC06953J-s001.pdf]

Supplementary Information (SI) for Chemical Science.
This journal is © The Royal Society of Chemistry 2026

Supporting Information

Room-Temperature Superprotonic Conductivity in COOH-Functionalized Multicomponent Covalent Organic Frameworks

Gouri Chakraborty,[a] Prasenjit Das,*[b] Biswajit Bhattacharya,*[a] Carsten Prinz[a], Franziska Emmerling,*[a] Arne Thomas*[b]

[a] BAM Federal Institute for Materials Research and Testing, Richard-Willstätter-Str. 11, 12489 Berlin, Germany
[b] Department of Chemistry, Functional Materials, Technische Universität Berlin, 10623 Berlin, Germany

E-mail: arne.thomas@tu-berlin.de

Section S1. General Materials and Methods

Materials: All purchased chemicals were used without further purification except where otherwise noted. *o*-DCB (1,2-Dichlorobenzene, 99%) anhydrous *n*-BuOH (*n*-Butanol, 99%), benzyl alcohol (BA), *t*-butyl alcohol (TBA), benzyl amine and styrene were purchased from Sigma Aldrich Chemicals. Tta (2,4,6-Tris(4- aminophenyl)triazine, 95%), Tpa-CHO (1,3,5-Tris(4-formylphenyl)triazine, >96.0%), were all supplied by TCI. Acetic acid (>99.0%), methanol (MeOH), Isopropanol (IPA), Ethanol (EtOH), were purchased from Carl Roth. All the substrates required for photocatalytic chemical conversion are purchased from TCI Germany.

X-ray Powder Diffraction patterns were collected on a Bruker D8 Advance diffractometer in reflection geometry operating with a Cu Kα anode (λ = 1.54178 Å) operating at 40 kV and 40 mA. Samples were ground and mounted as loose powders onto a Si sample holder. PXRD patterns were collected from 2 to 60 2θ degrees with a step size of 0.02 degrees and an exposure time of 2 seconds per step.

^{1}H NMR Spectra for the samples dissolved in suitable solvents were carried on Bruker Avance II 200 MHz. ^{13}C Solid-state NMR (cross polarization magic-angle spinning (CP/MAS)) spectra were carried out on a Bruker Avance 400 MHz spectrometer operating at 100.6 MHz.

Thermogravimetric Analyses (TGA) were performed using a TGA Q500 thermal analysis system under a N_2 atmosphere from room temperature to 800 °C at a ramping rate of 1 °C /min.

Attenuated Total Reflectance Fourier-Transform Infrared Spectrometry (ATR-FT-IR) was conducted using a PerkinElmer Spectrum Two spectrometer with diamond/ZnSe ATR accessory. All spectra were collected using a $LiTaO_3$ MIR detector over a range of 450 to 4000 cm^{-1}. All spectra were processed using Spectrum 10 software.

Solid-State Diffuse Reflectance Ultraviolet–visible Spectroscopy (UV-vis) spectra of the pristine COF powders and starting monomers have been collected on Varian Cary 300 UV-vis Spectrophotometer.

Nitrogen Sorption Measurement was performed at 77 K using an Autosorb-iQ-MP from Quantachrome. Prior to the analysis the sample was activated at 120 °C for 24 h. Using the N_2 adsorption isotherm, the surface area was calculated over a pressure range 0.05-0.1 = p/p_0 using Brunauer-Emmett-Teller (BET) methods.

Field Emission Scanning Electron Microscopy (FESEM) was measured on a ZEISS GeminiSEM500. **PMCR-1** was observed directly without gold coating in nanoVP mode.

High Resolution Transmission Electron Microscopy (HRTEM) Transmission electron microscopy (TEM) imaging was conducted using a Talos F200S Microscope (Thermo Fisher Scientific) operated at an accelerating voltage of 200 kV. This technique involves the transmission of a high-energy electron beam through a specimen to generate detailed images. Images were acquired in TEM mode using a Ceta 16M camera. Specimen preparation involved depositing 10 µL of sample solution (1 mg of sample dissolved in 0.5 mL EtOH and 0.5 mL acetone) onto a 3 mm copper grid (lacey, 400 mesh) and allowing it to air dry at room temperature. Imaging and data analysis were performed using Velox software (version 3.3).

Proton Conduction Measurement

Proton conductivities of all COFs were measured by alternative-current (AC) impedance measurements using an impedance and gain-phase analyzer (Biologic SP150e) over a frequency range from 10 mHz to 1 MHz with an input voltage amplitude of 10 mV. Powder samples were pelletized under a pressure of 4-ton cm^{-2} for 5 min. Each pellet was coated by silver paste on both sides and dried in air overnight. For each sample, a compacted pellet was produced with a diameter of 5.0 mm and thickness of 0.5 mm for all COFs. Then the corresponding proton conductivities of all COFs were measured by inserting the pellet in a stainless-steel cell (home-made) with the two-electrode setup. The measurements were carried out over a large range of temperatures (25–90 °C) at a constant relative humidity (98%) and relative humidity levels (68–98% RH) at a constant temperature (25°C). A programmable Binder constant climate chamber (Model KMF) was used to keep the sample at the desired temperature and relative humidity for proton conductivity measurement. The conditions of the chamber for each measurement were kept at equilibrium for 12 h. The conductivities were calculated from the first semicircle of the Nyquist plots of impedance spectra by fitting with the most suitable equivalent circuit. Proton conductivity was calculated using the following equation

$$\sigma = l \,/\, AZ$$

where l is the distance between the electrodes, which is equal to the thickness of the pellet and A is the cross section of the pellet. Z is the bulk resistance of the sample which was extracted from the impedance plots.

Synthesis of Model Compound

A Pyrex glass tube (10 mL) was charged with 3,4-dimethoxyaniline (100 mg, 0.17 mmol), 4-fluorobenzaldehyde (60 mg, 0.17 mmol), triethylamine (0.6 mmol, 60 μL), and ammonium iodide (NH_4I) (2.7 mg, 0.01 mmol), in ethanol (5 mL). The tube was first sonicated for 30 minutes to form bulk solid and then flash frozen at 77 K (liquid N_2 bath) and degassed by three times of freeze-pump-thaw cycles. The internal pressure was evacuated to 10^{-3} mbar. The tube was sealed and heated at 120 °C for 1 day. The formed crystal like solid was filtered and gently washed with THF and MeOH. Finally, the powder was dried and utilized for characterization. Product was isolated as off-yellow powder = 101 mg.

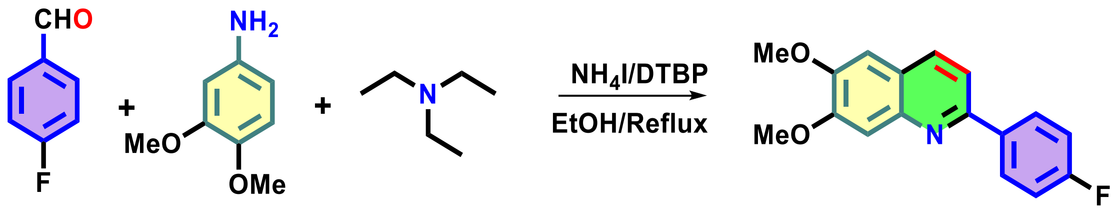

Scheme S1. Synthesis of the model compound**.**

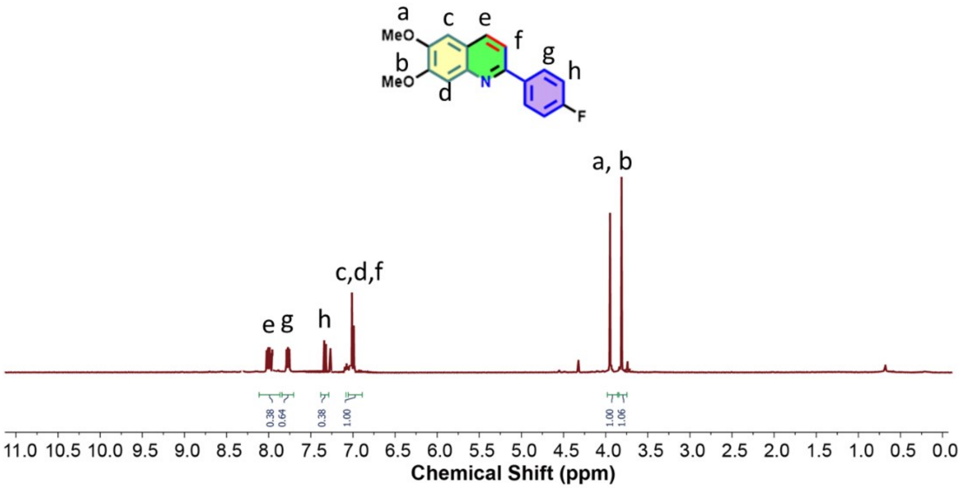

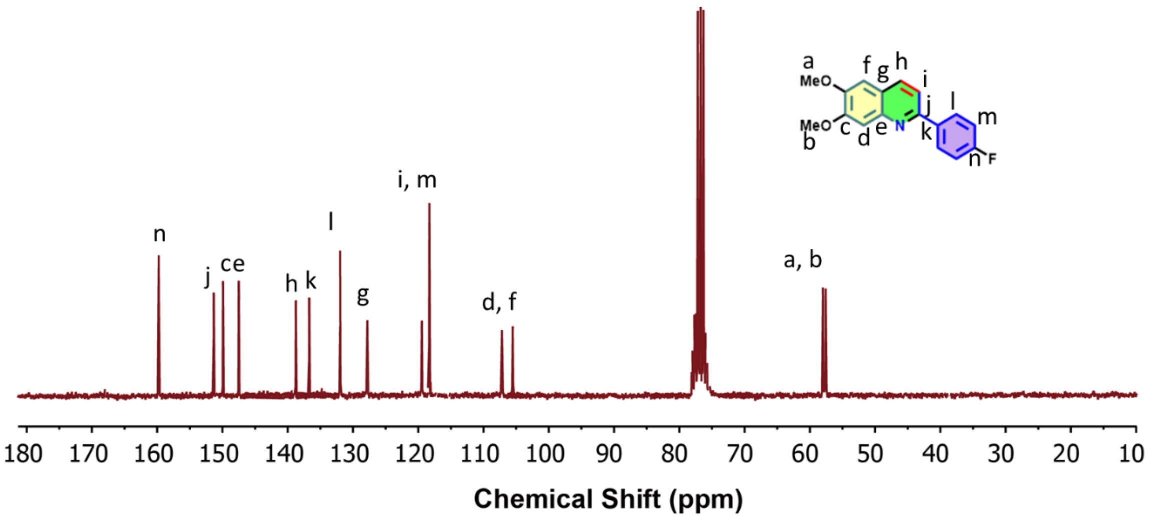

Fig. S1 ^{1}H-NMR (top) and ^{13}C-NMR (bottom) spectra of the model compound in $CDCl_3$.

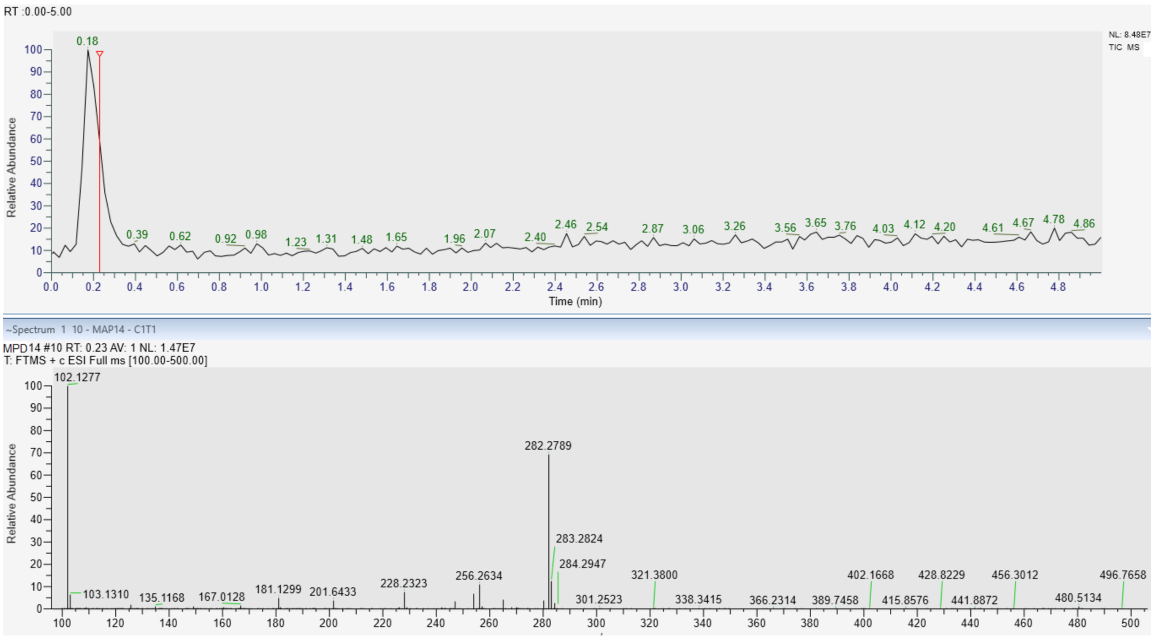

Fig. S2 HRMS spectra of the model compound [M+H: 283.28].

Synthesis of Qy-COOH

A Pyrex glass tube (15 mL) was charged with 2,4,6-Tris(4-aminophenyl)benzene (55 mg, 0.17 mmol), 4,4',4''-(1,3,5-triazine-2,4,6-triyl)tribenzaldehyde (60 mg, 0.17 mmol), pyruvic acid (PA) (0.6 mmol, 60 μL), 2,3-dichloro-5,6-dicyano-1,4-benzoquinone (DDQ) (5 mg, 0.04 mmol), 1.5 mL *o*-DCB and 1.5 mL *n*-BuOH. The tube was first sonicated for 20 minutes and then flash frozen at 77 K (liquid N_2 bath) and degassed by three times of freeze-pump thaw cycles. The internal pressure was evacuated to 10^{-3} mbar. The tube was sealed and placed in a preheated oven at 120 °C for 3 days. After finishing heating, the tube was cooled down and cut. The solid

was washed one time with acetone, transferred to a filter paper and purified by Soxhlet extraction (solvent mixture of MeOH, Acetone, THF and Ethanol) for 12 h. Finally, the powder was dried in a normal oven at 80 °C. Yield = 92% (101 mg, brownish green color).

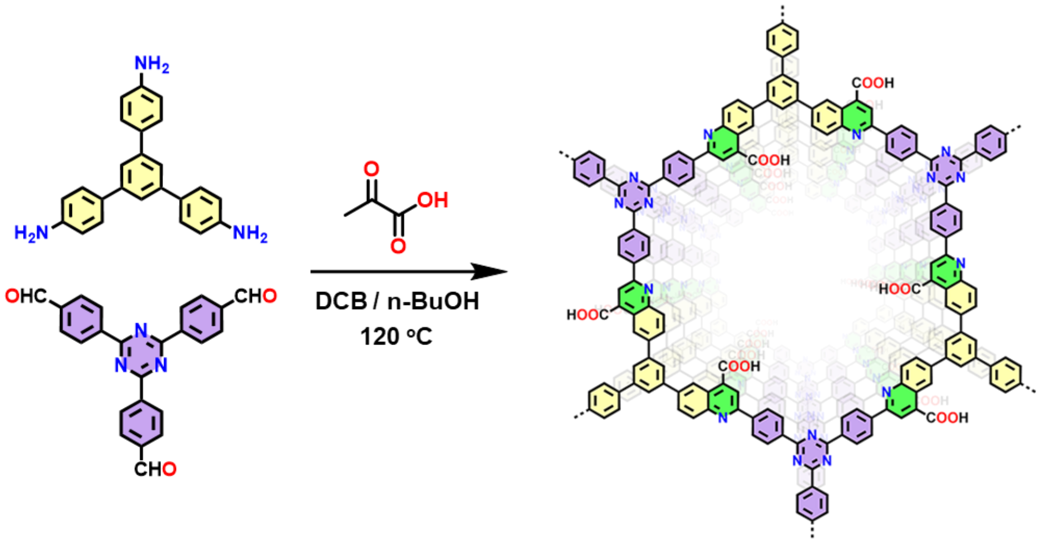

Scheme S2. Synthesis of **Qy-COOH**.

Synthesis of Qy-H

A Pyrex glass tube (15 mL) was charged with 2,4,6-Tris(4-aminophenyl)benzene (55 mg, 0.17 mmol), 4,4',4"-(1,3,5-triazine-2,4,6-triyl)tribenzaldehyde (60 mg, 0.17 mmol), ammonium iodide (34.8 mg, 0.24 mmol), di-*tert*-butyl peroxide (45 µL, 0.24 mmol), triethylamine (67 µL, 0.36 mmol), 2,3-dichloro-5,6-dicyano-1,4-benzoquinone (DDQ) (5 mg, 0.04 mmol), 1.5 mL *o*-DCB and 1.5 mL *n*-BuOH. The tube was first sonicated for 20 minutes and then flash frozen at 77 K (liquid N_2 bath) and degassed by three times of freeze-pump thaw cycles. The internal pressure was evacuated to 10^{-3} mbar. The tube was sealed and placed in a preheated oven at 120 °C for 3 days. After finishing heating, the tube was cooled down and cut. The solid was washed one time with acetone, transferred to a filter paper and purified by Soxhlet extraction (solvent mixture of MeOH, Acetone, THF and Ethanol) for 12 h. Finally, the powder was dried in a normal oven at 80 °C. Yield = 93% (108 mg, green color).

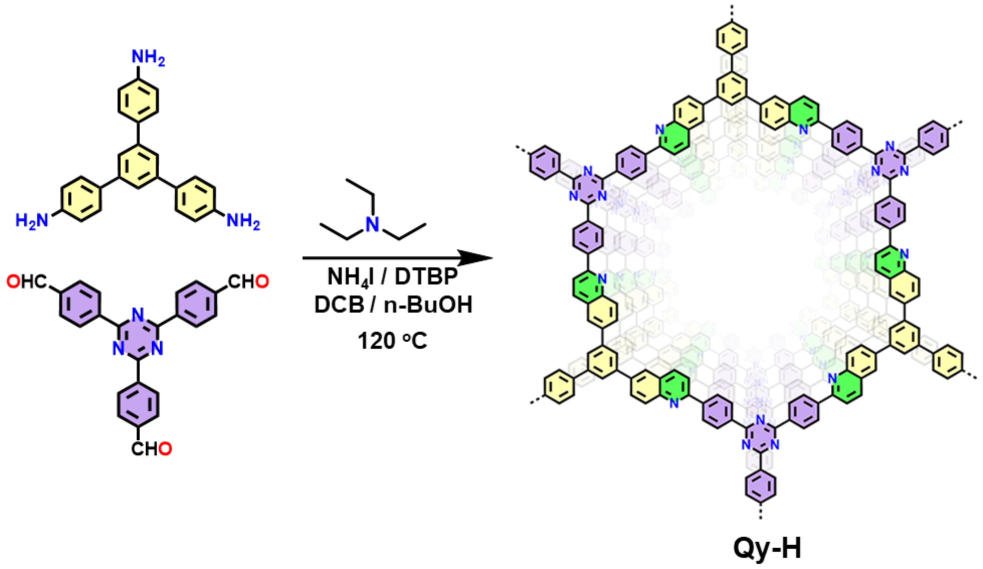

Scheme S3. Synthesis of **Qy-H**.

Synthesis of Im-1

The synthesis of **Im-1** has been carried out following a previously published paper.[1]

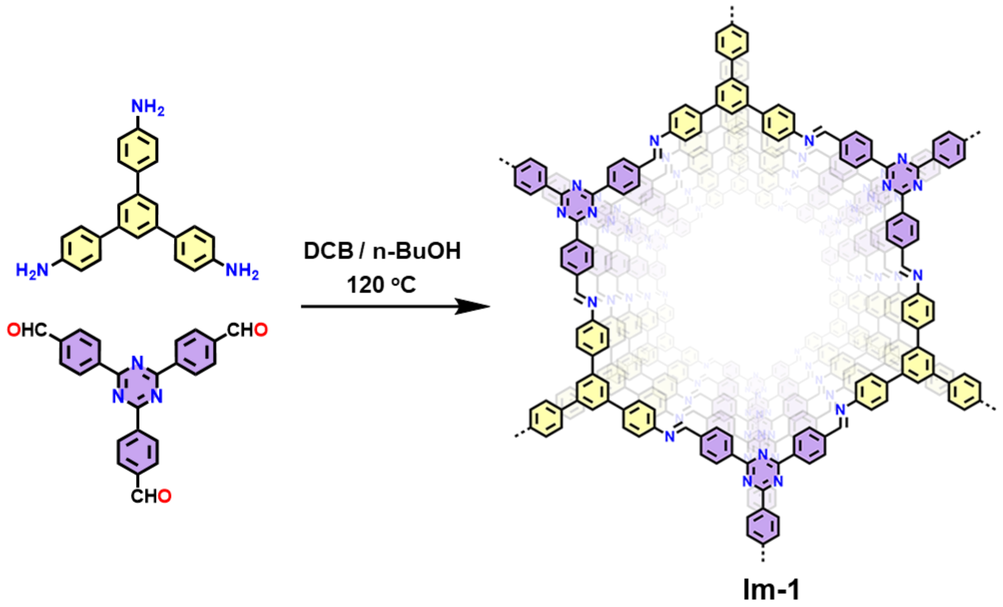

Scheme S4. Synthesis of **Im-1**.

Synthesis of Qy-COOH1

A Pyrex glass tube (15 mL) was charged with 2,4,6-Tris(4-aminophenyl)triazine (60 mg, 0.16 mmol), 4,4',4''-(amino-2,4,6-triyl)tribenzaldehyde (56 mg, 0.16 mmol), pyruvic acid (PA) (0.6 mmol, 60 μL), 2,3-dichloro-5,6-dicyano-1,4-benzoquinone (DDQ) (5 mg, 0.04 mmol), 1.5 mL *o*-DCB and 1.5 mL *n*-BuOH. The tube was first sonicated for 30 minutes to form bulk solid and then flash frozen at 77 K (liquid N_2 bath) and degassed by three times of freeze-pump-thaw cycles. The internal pressure was evacuated to 10^{-3} mbar. The tube was sealed and heated at 120 °C for 3 days. The tube was allowed to cool to room temperature, the solid was removed from the tube with acetone and washed with methanol until the filtrate was colourless. The solid was washed one time with acetone, transferred to a filter paper and purified by Soxhlet extraction (solvent mixture of MeOH, Acetone, THF and Ethanol) for 12 h. Finally, the powder was dried in a normal oven at 80 °C. Yield = 84% (123 mg).

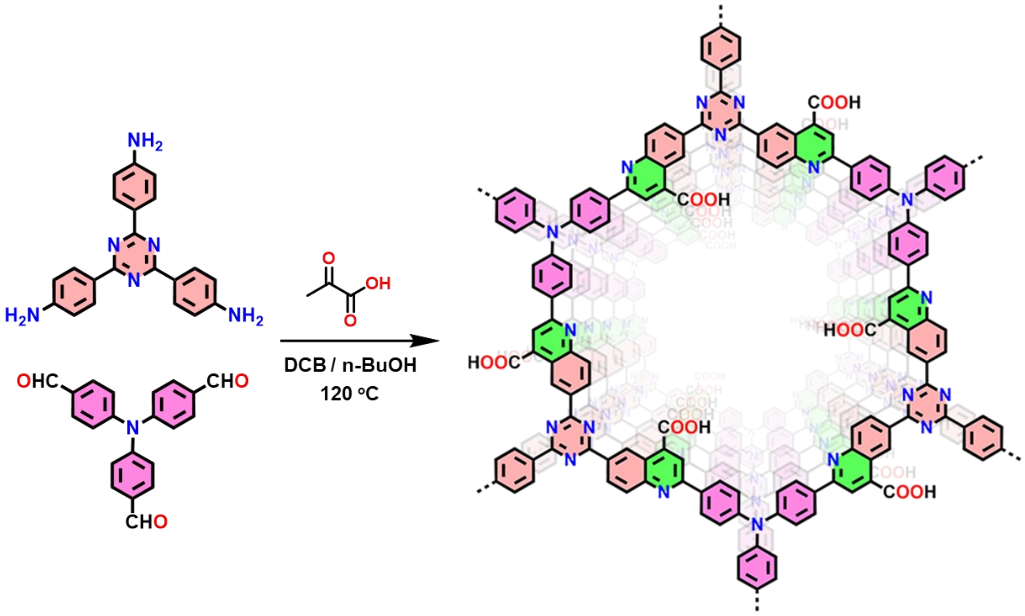

Scheme S5. Synthesis of **Qy-COOH1**.

Synthesis of OH-Qy-COOH

A Pyrex glass tube (15 mL) was charged with 2,4,6-Tris(4-aminophenyl)triazine (60 mg, 0.16 mmol), 5'-(4-Formyl-3-hydroxyphenyl)-3,3''-dihydroxy-[1,1':3',1''-terphenyl]-4,4''-dicarbaldehyde (75 mg, 0.16 mmol), pyruvic acid (PA) (0.6 mmol, 60 μL), 2,3-dichloro-5,6-dicyano-1,4-benzoquinone (DDQ) (5 mg, 0.04 mmol), 1.5 mL *o*-DCB and 1.5 mL *n*-BuOH. The tube was first sonicated for 30 minutes to form bulk solid and then flash frozen at 77 K (liquid N_2 bath) and degassed by three times of freeze-pump-thaw cycles. The internal pressure was evacuated to 10^{-3} mbar. The tube was sealed and heated at 120 °C for 3 days. The tube was allowed to cool to room temperature, the solid was removed from the tube with acetone and washed with methanol until the filtrate was colourless. The solid was washed one time with acetone, transferred to a filter paper and purified by Soxhlet extraction (solvent mixture of MeOH, Acetone, THF and Ethanol) for 12 h. Finally, the orange powder was dried in a normal oven at 80 °C. Yield = 84% (123 mg).

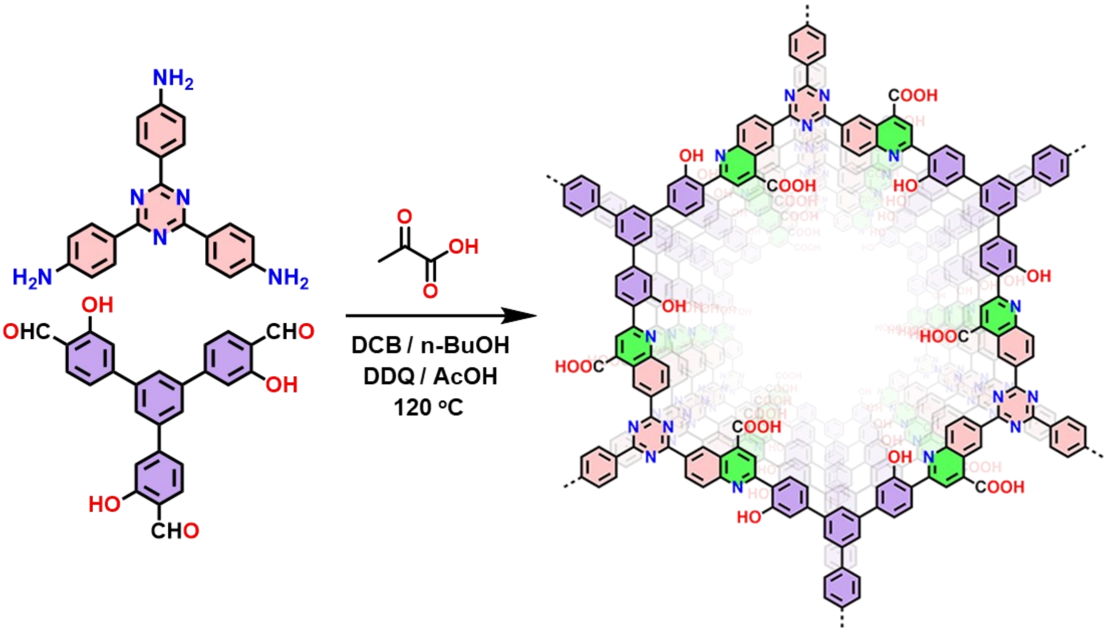

Scheme S6. Synthesis of **OH-Qy-COOH**.

Synthesis of OH-Qy-H

A Pyrex glass tube (15 mL) was charged with 2,4,6-Tris(4-aminophenyl)benzene (55 mg, 0.17 mmol), 5'-(4-Formyl-3-hydroxyphenyl)-3,3''-dihydroxy-[1,1':3',1''-terphenyl]-4,4''-dicarbaldehyde (75 mg, 0.16 mmol), ammonium iodide (34.8 mg, 0.24 mmol), di-*tert*-butyl peroxide (45 µL, 0.24 mmol), triethylamine (67 µL, 0.36 mmol), 2,3-dichloro-5,6-dicyano-1,4-benzoquinone (DDQ) (5 mg, 0.04 mmol), 1.5 mL *o*-DCB and 1.5 mL *n*-BuOH. The tube was first sonicated for 20 minutes and then flash frozen at 77 K (liquid N_2 bath) and degassed by three times of freeze-pump thaw cycles. The internal pressure was evacuated to 10^{-3} mbar. The tube was sealed and placed in a preheated oven at 120 °C for 3 days. After finishing heating, the tube was cooled down and cut. The solid was washed one time with acetone, transferred to a filter paper and purified by Soxhlet extraction (solvent mixture of MeOH, Acetone, THF and Ethanol) for 12 h. Finally, the yellow brown powder was dried in a normal oven at 80 °C. Yield = 83% (112 mg).

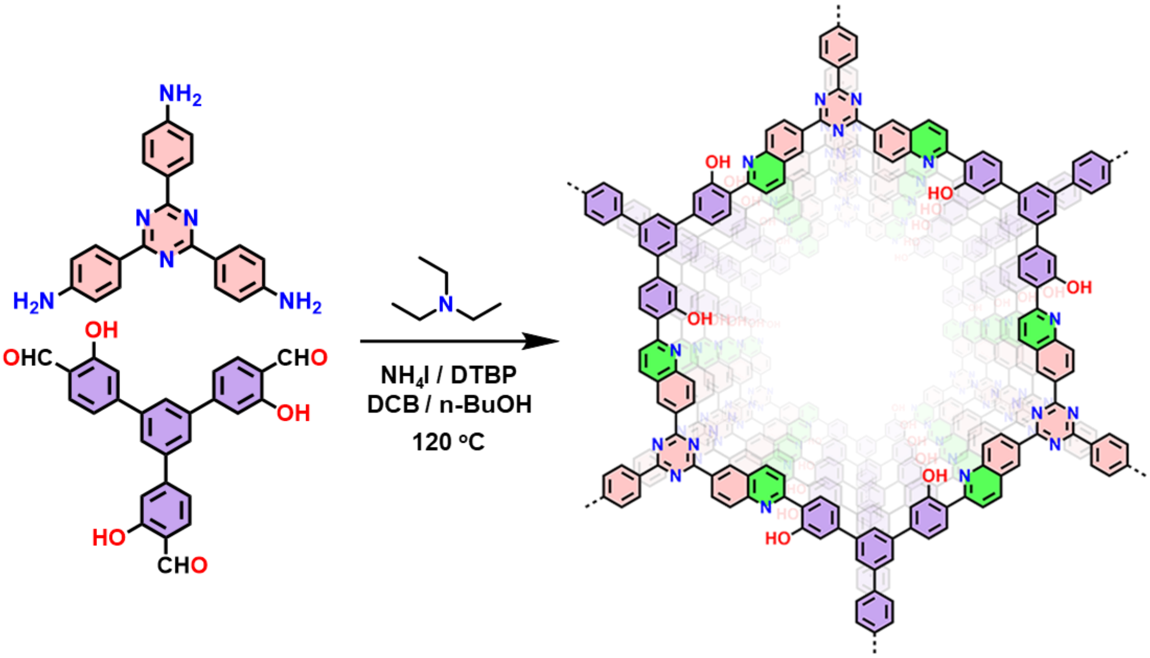

Scheme S7. Synthesis of **OH-Qy-H**.

Synthesis of OH-Im

A Pyrex glass tube (15 mL) was charged with 2,4,6-Tris(4-aminophenyl)benzene (55 mg, 0.17 mmol), 5'-(4-Formyl-3-hydroxyphenyl)-3,3''-dihydroxy-[1,1':3',1''-terphenyl]-4,4''-dicarbaldehyde (75 mg, 0.16 mmol), 100 μL AcOH (6M), 1.5 mL *o*-DCB and 1.5 mL *n*-BuOH. The tube was first sonicated for 20 minutes and then flash frozen at 77 K (liquid N_2 bath) and degassed by three times of freeze-pump thaw cycles. The internal pressure was evacuated to 10^{-3} mbar. The tube was sealed and placed in a preheated oven at 120 °C for 3 days. After finishing heating, the tube was cooled down and cut. The solid was washed one time with acetone, transferred to a filter paper and purified by Soxhlet extraction (solvent mixture of MeOH, Acetone, THF and Ethanol) for 12 h. Finally, the yellow powder was dried in a normal oven at 80 °C. Yield = 86% (108 mg, yellow color).

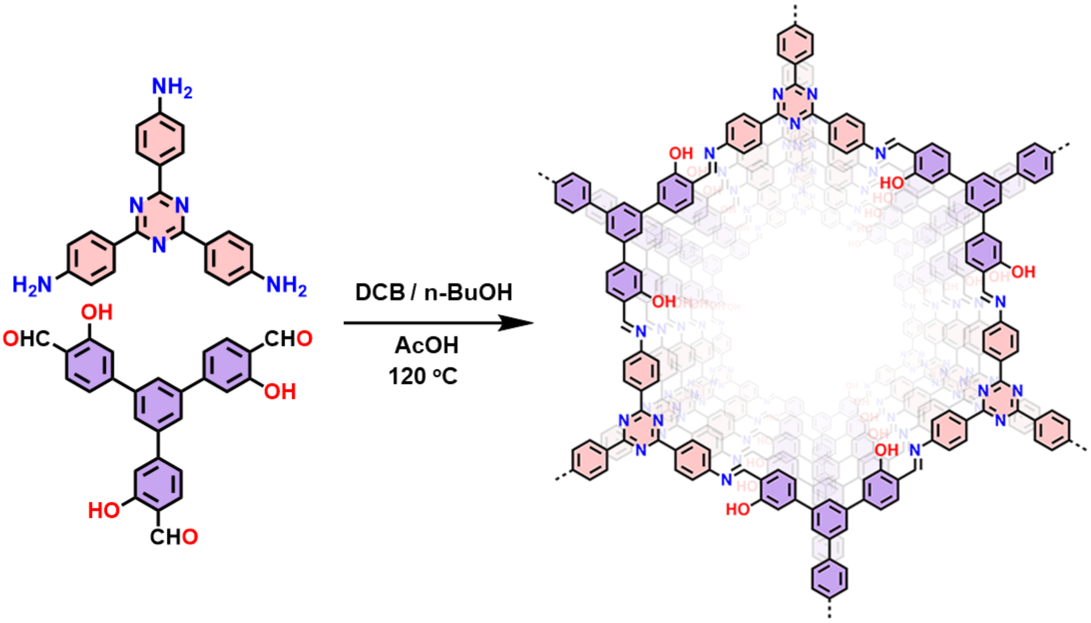

Scheme S8. Synthesis of **OH-Im**.

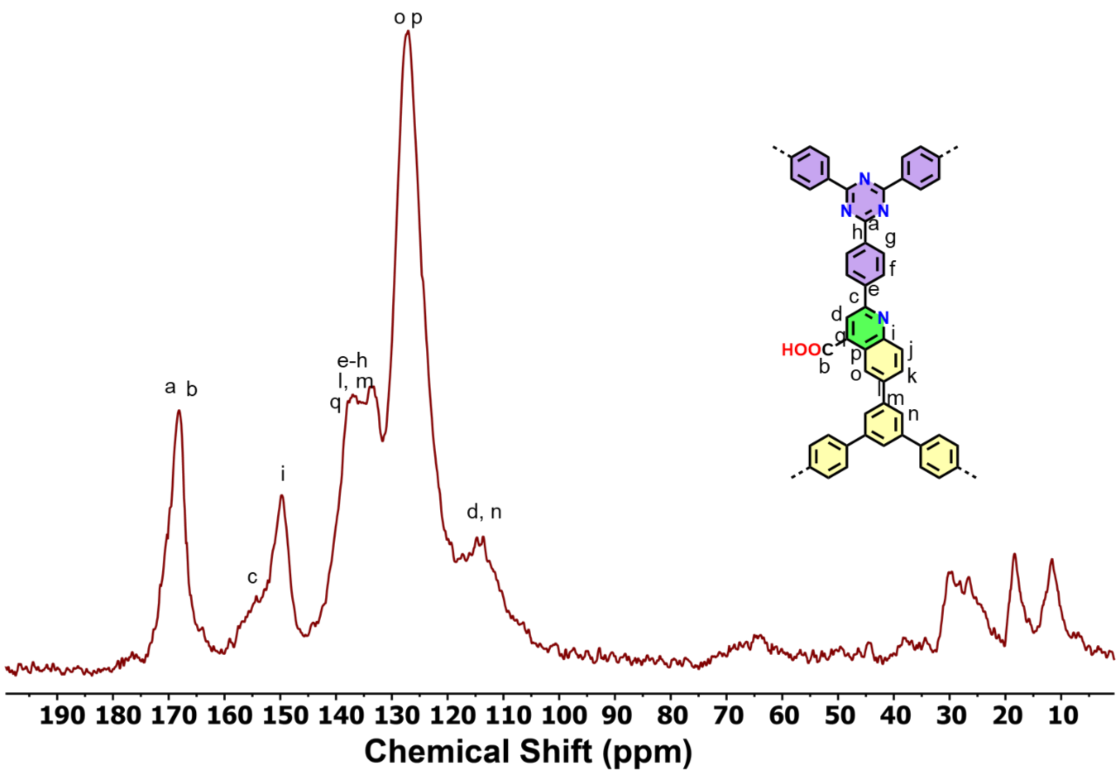

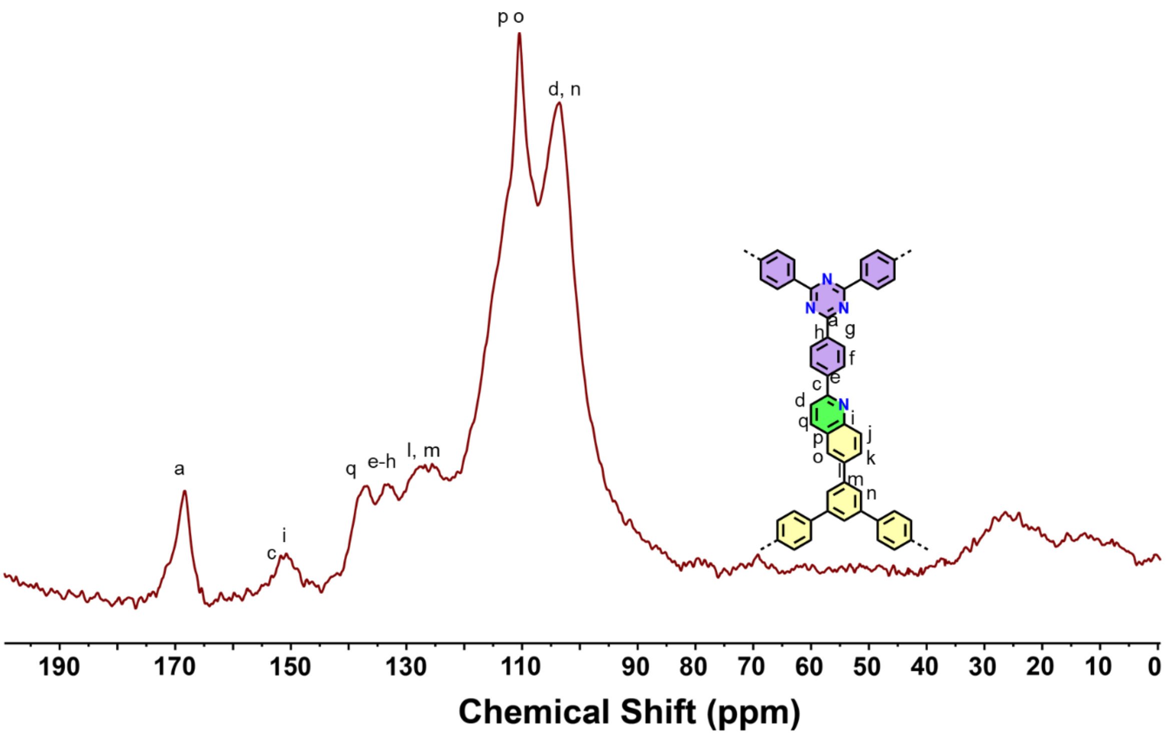

Fig. S3 CP-MAS ^{13}C-NMR spectra of **Qy-COOH** (top) and **Qy-H** (bottom). The peak near 0-35 ppm corresponding to spinning sidebands.

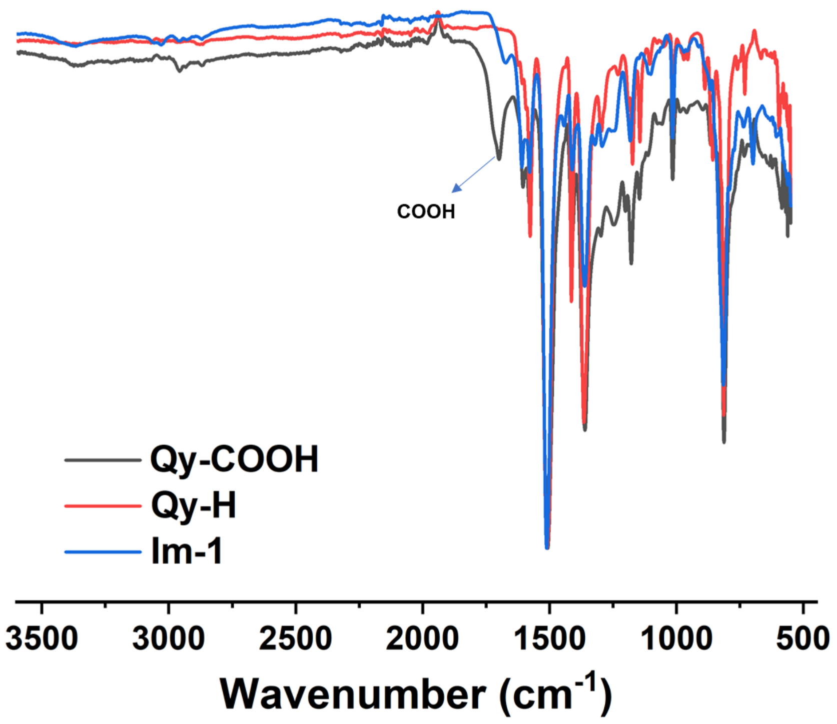

Fig. S4 FTIR spectra of **Qy-COOH, Qy-H** and **Im-1**.

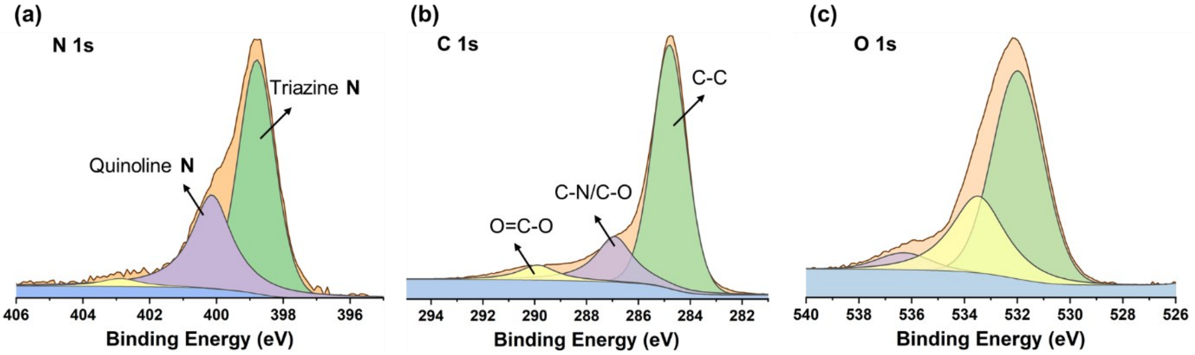

Fig. S5 (a) N(1s), (b) C(1s) and (c) O1s XPS spectra of **Qy-COOH**.

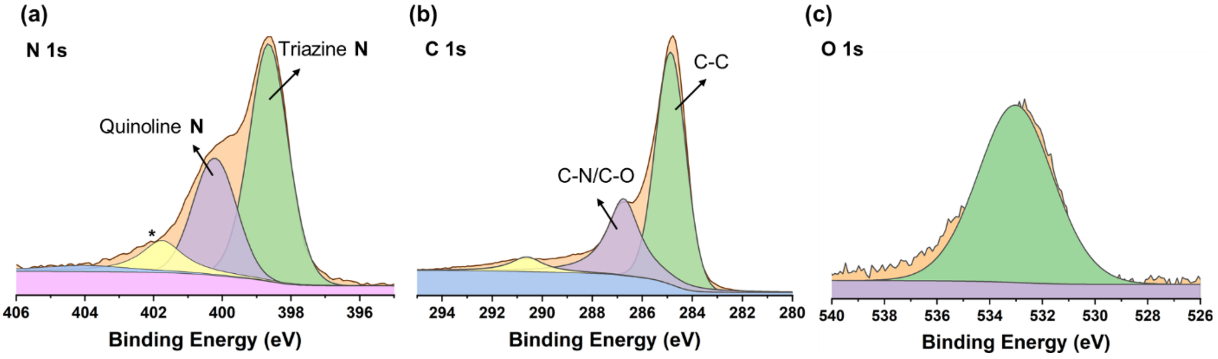

Fig. S6 (a) N(1s) (asterisk for surface oxidation), (b) C(1s) and (c) O1s XPS spectra of **Qy-H**. Note: The small O 1s signal likely arises from surface adsorption of atmospheric oxygen, minor surface oxidation, and possibly trace amounts of unreacted aldehyde end groups.

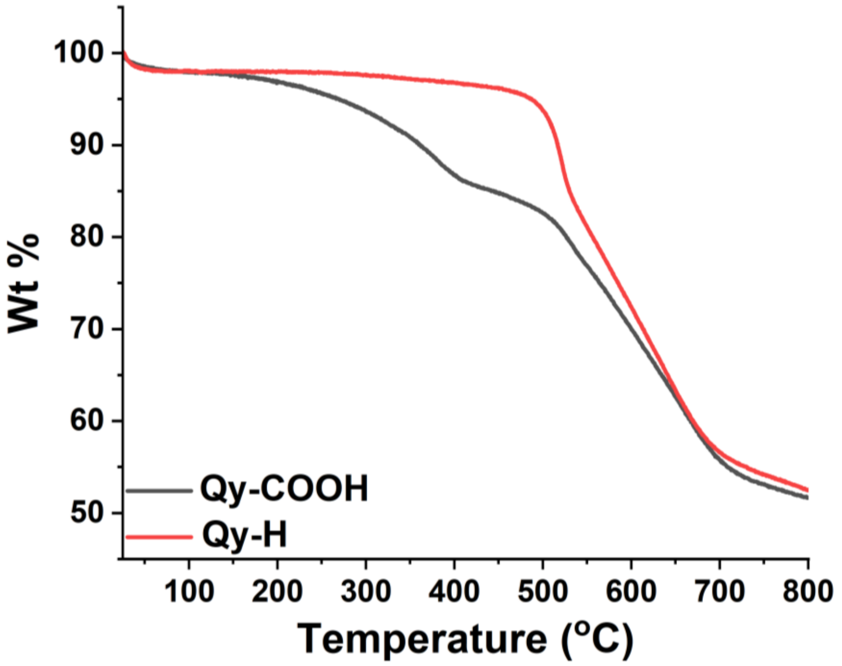

Fig. S7 TGA profiles of **Qy-COOH** and **Qy-H**.

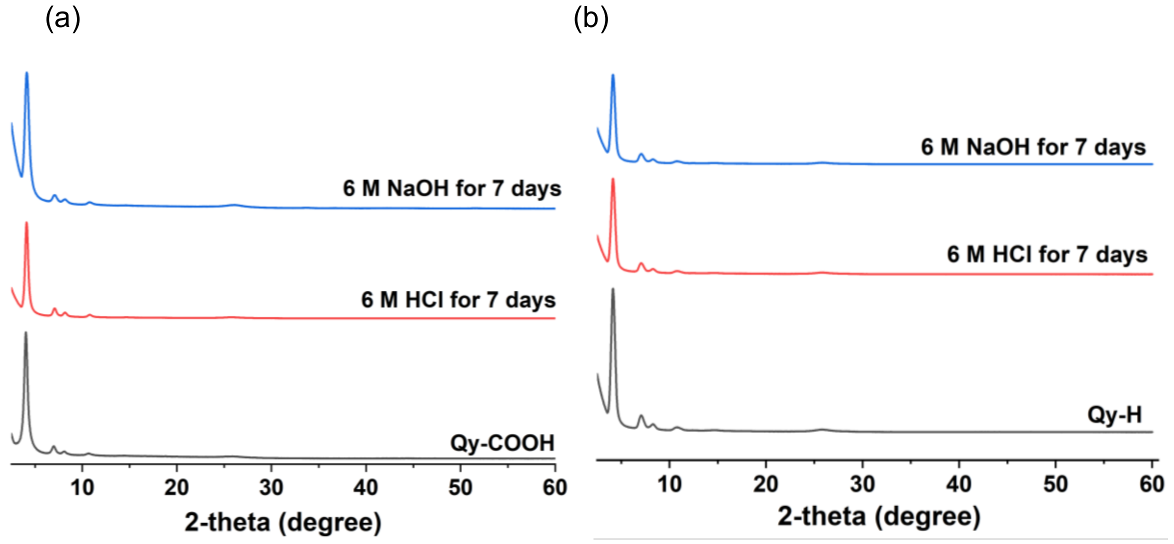

Fig. S8 Stability study of (a) **Qy-COOH** and (b) **Qy-H** in presence of aq. 6 M HCL and aq. 6 M NaOH for 7 days.

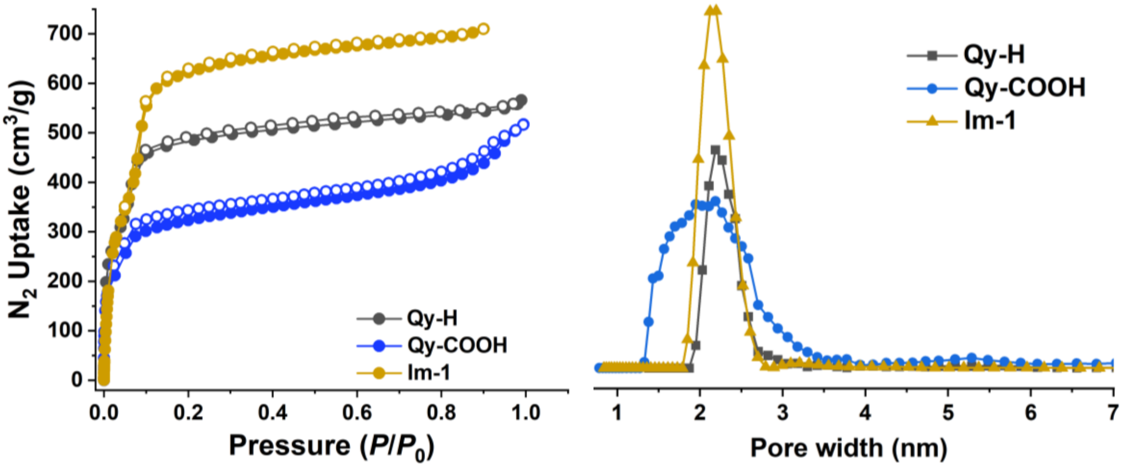

Fig. S9 (Left) N_2 sorption isotherms of **Qy-COOH, Qy-H** and **Im-1**. (Right) Pore size distribution of **Qy-COOH**, **Qy-H** and **Im-1**.

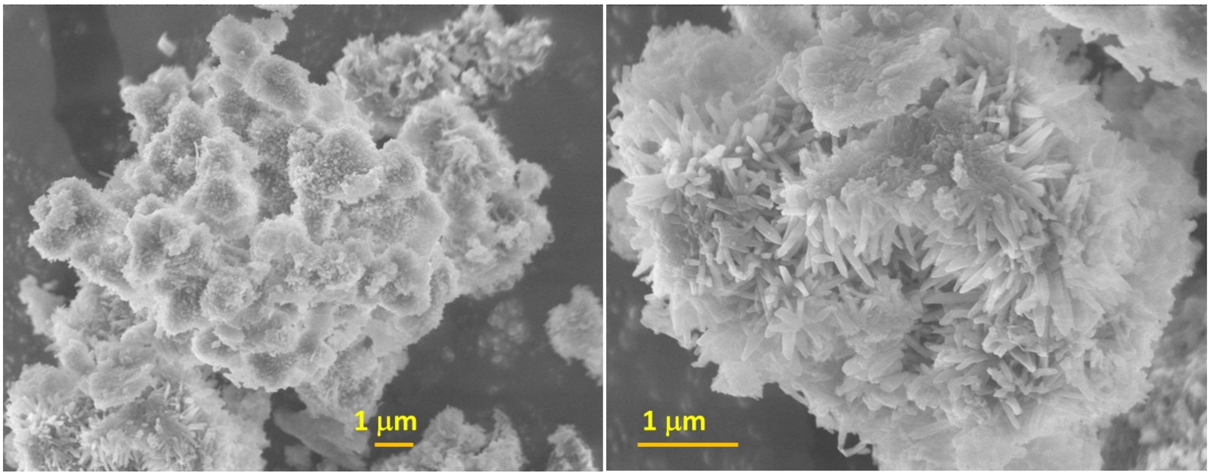

Fig. S10 FESEM images of **Qy-COOH**.

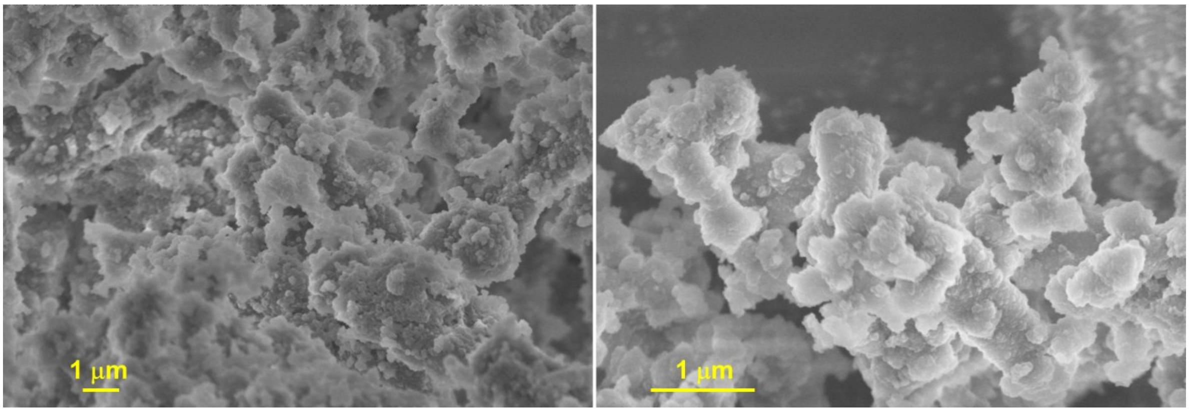

Fig. S11 FESEM images of **Qy-H**.

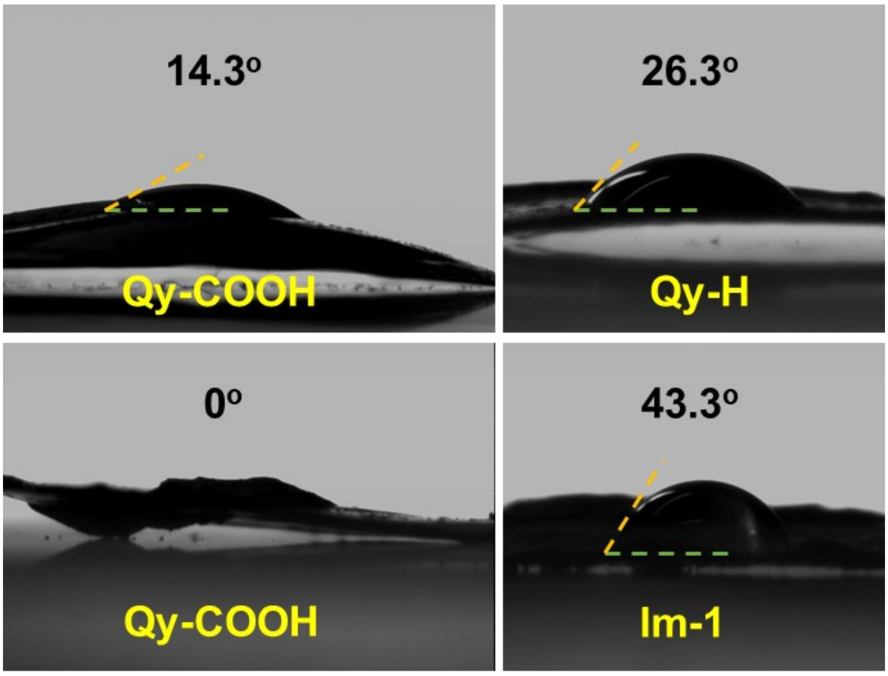

Fig. S12 Water contact angle measurements of **Qy-COOH**, **Qy-H** and **Im-1**. In case of **Qy-COOH** all water being adsorbed after few seconds.

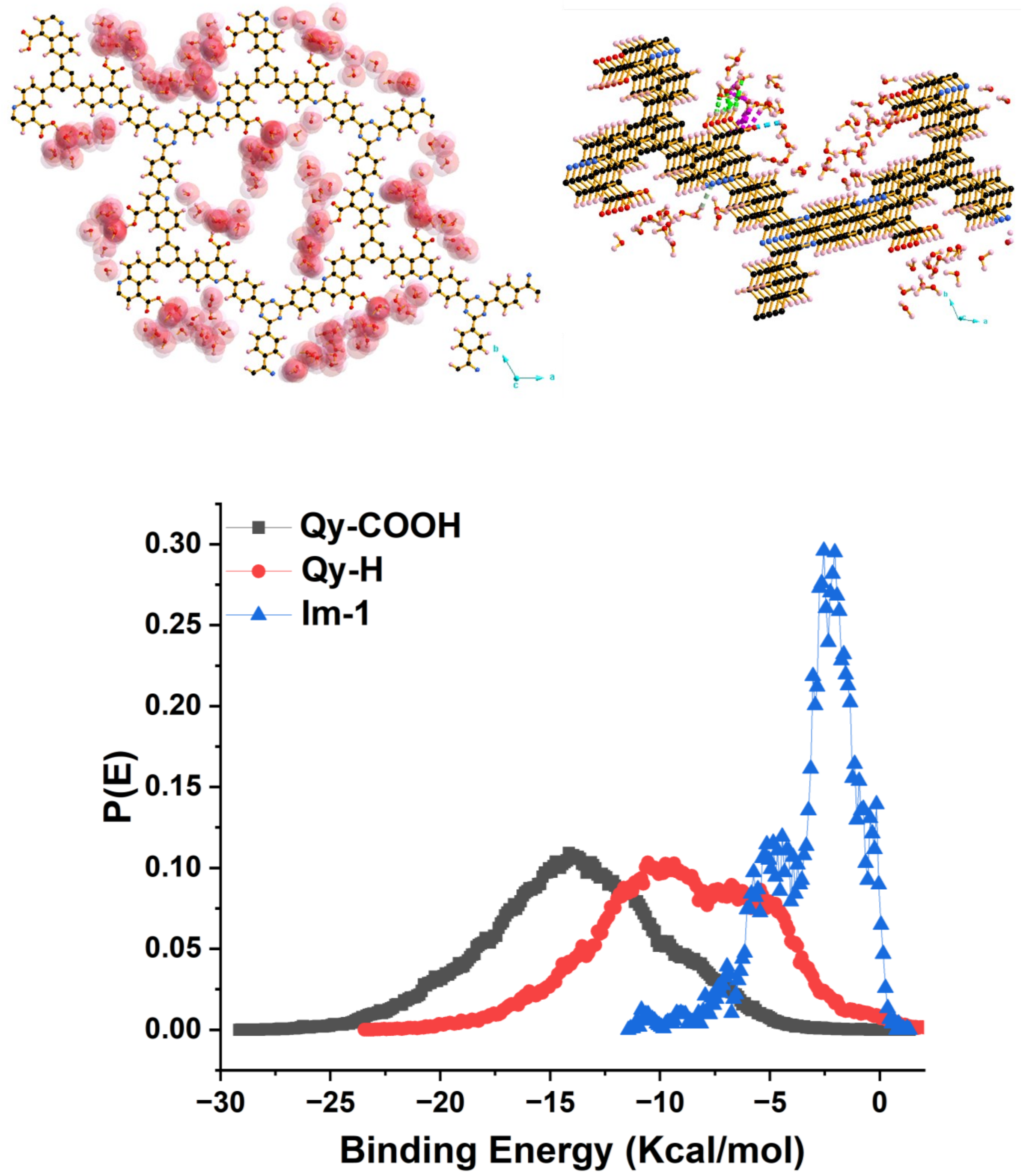

Fig. S13 CBMC molecular simulation: adsorption position and probability distribution plot of H_2O at 0.1 bar in **Qy-COOH**. Binding energy of H_2O in presence of **Qy-COOH**, **Qy-H** and **Im-1**.

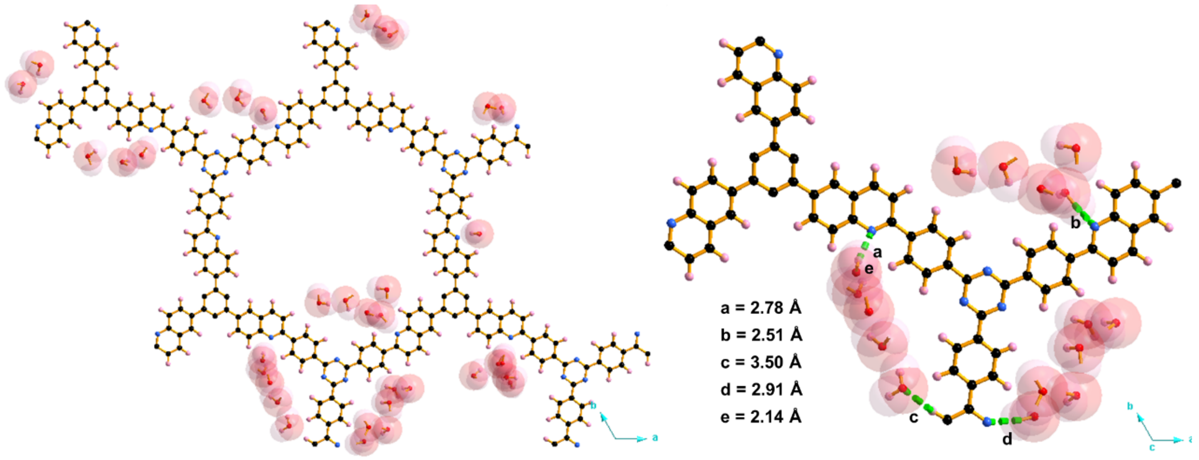

Fig. S14 CBMC molecular simulation: adsorption position and probability distribution plot of H_2O at 0.1 bar in **Qy-H**.

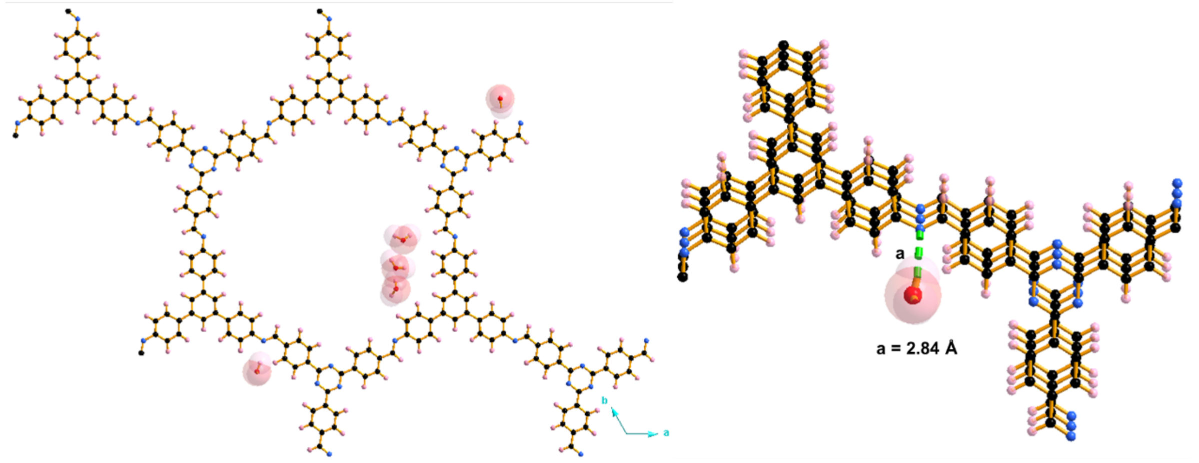

Fig. S15 CBMC molecular simulation: adsorption position and probability distribution plot of H_2O at 0.1 bar in **Im-1**.

Stepwise Synthesis of **Qy-COOH** from **Im-1** via Post synthetic modification:

A Pyrex glass tube (15 mL) was charged with **Im-1** (60 mg), pyruvic acid (PA) (0.6 mmol, 60 μL), 2,3-dichloro-5,6-dicyano-1,4-benzoquinone (DDQ) (5 mg, 0.04 mmol), 1.5 mL *o*-DCB and 1.5 mL *n*-BuOH. The tube was first sonicated for 20 minutes and then flash frozen at 77 K (liquid N_2 bath) and degassed by three times of freeze-pump thaw cycles. The internal pressure was evacuated to 10^{-3} mbar. The tube was sealed and placed in a preheated oven at 120 °C for 2 days. After finishing heating, the tube was cooled down and cut. The solid was washed one time with acetone, transferred to a filter paper and purified by Soxhlet extraction (solvent mixture of MeOH, Acetone, THF and Ethanol) for 12 h. Finally, the powder was dried in a normal oven at 80 °C. Yield = 52 mg, yellow-brownish color.

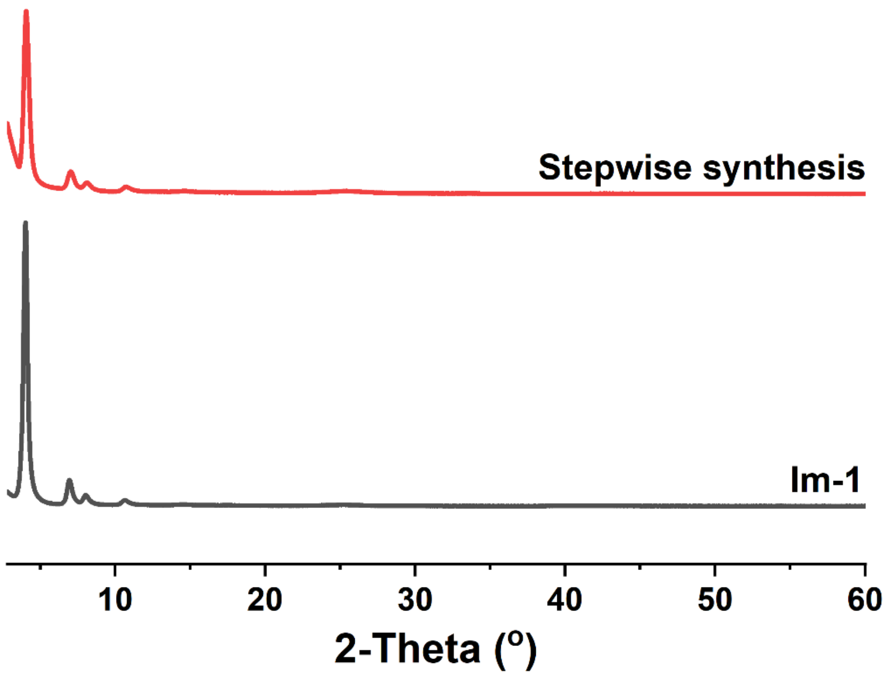

Fig. S16 PXRD patterns of step wise synthesis of **Qy-COOH.**

(a)

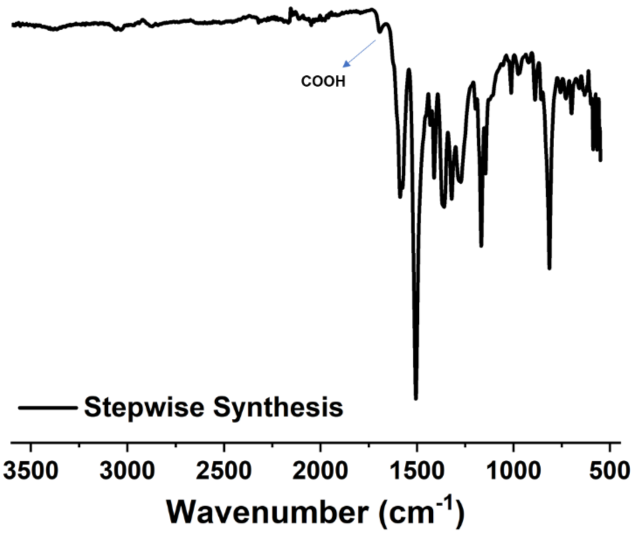

(b)

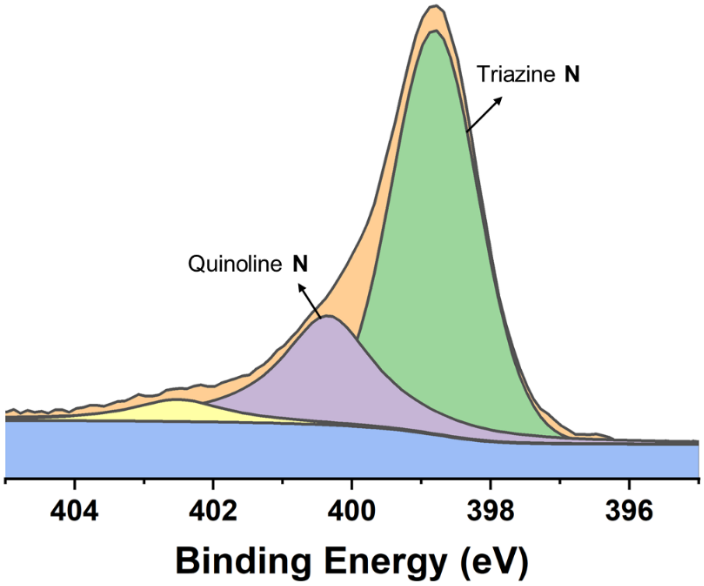

Fig. S17 (a) FTIR spectrum and (b) N(1s) XPS spectra of **Qy-COOH** prepared via PSM. The spectra show a lower conversion to quinoline linkages compared to the direct multicomponent reaction.

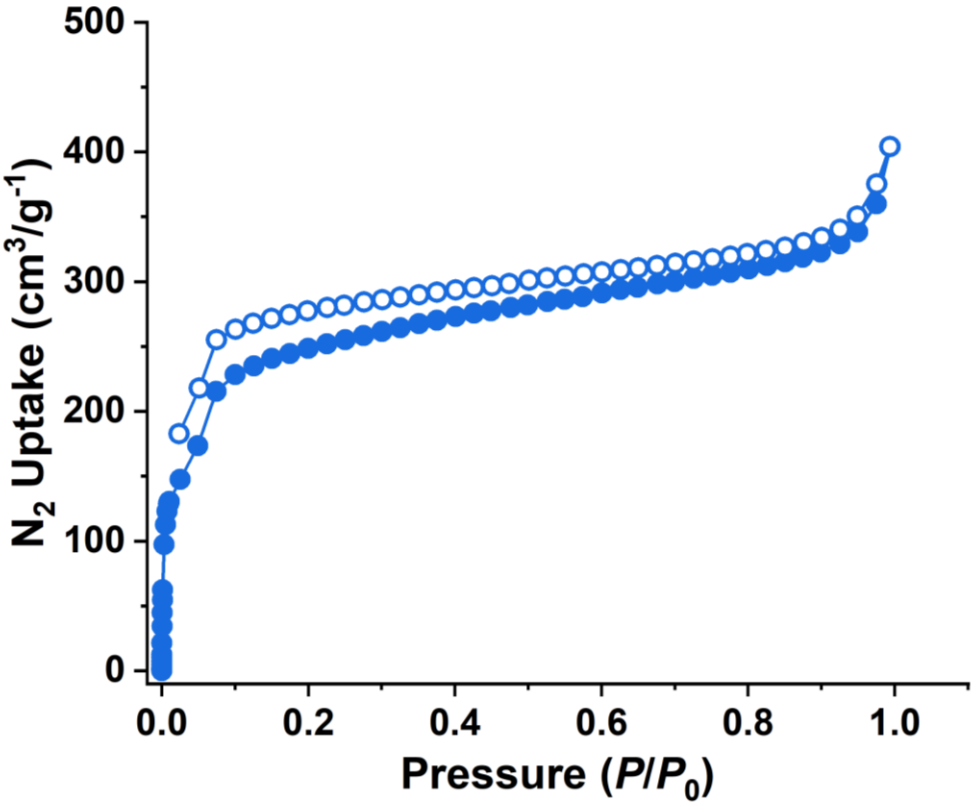

Fig. S18 N_2 sorption isotherm of **Qy-COOH** prepared via PSM**.** BET surface area: 605 m_2/g.

N.B: Both the crystallinity and BET surface area of **Qy-COOH** decreased when it is prepared via PSM, with less than 50% conversion of the imine linkages (FTIR and XPS analysis). These results highlight the significance of the multicomponent reaction approach. Additionally, these factors contribute to the lower proton conductivity observed in comparison to the multicomponent COF.

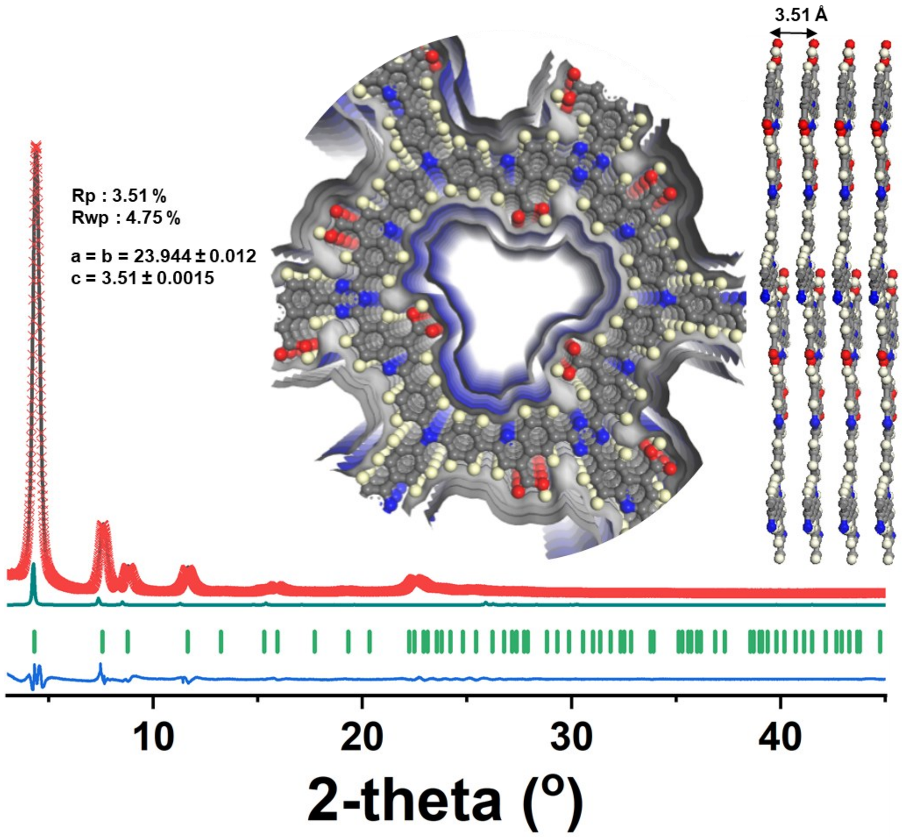

Fig. S19 Simulated and experimental PXRD patterns of **Qy-COOH1**. Experimental (black dotted line) and Pawley refined (red cross) patterns, Bragg position (green) and corresponding difference plot (blue line). The theoretical PXRD pattern for an eclipsed AA stacking model of **Qy-COOH1** is provided as cyan line. Top and side view of AA stacking in **Qy-COOH1**. (Color code: C, gray spheres; O, red spheres; N, blue spheres, H, pale yellow spheres).

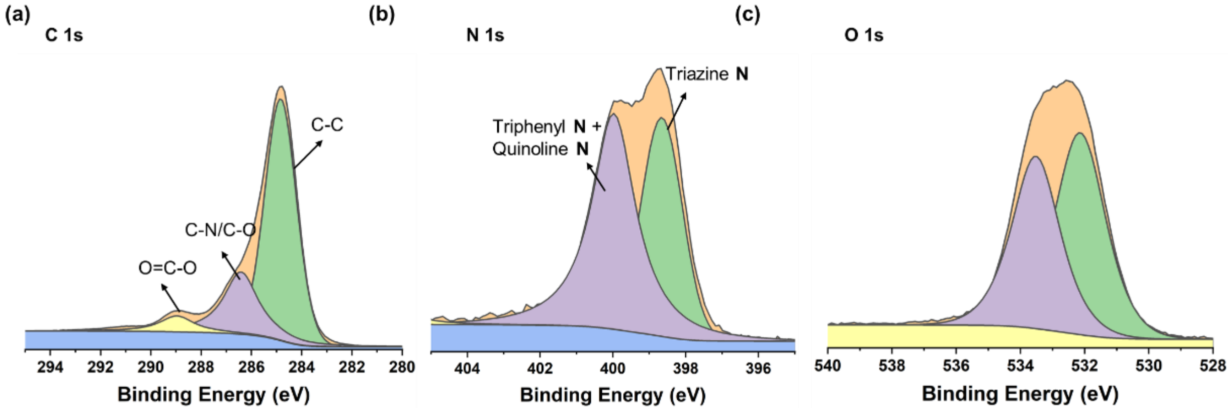

Fig. S20 (a) C1s, (b) N(1s) and (c) O1s XPS spectra of **Qy-COOH1.**

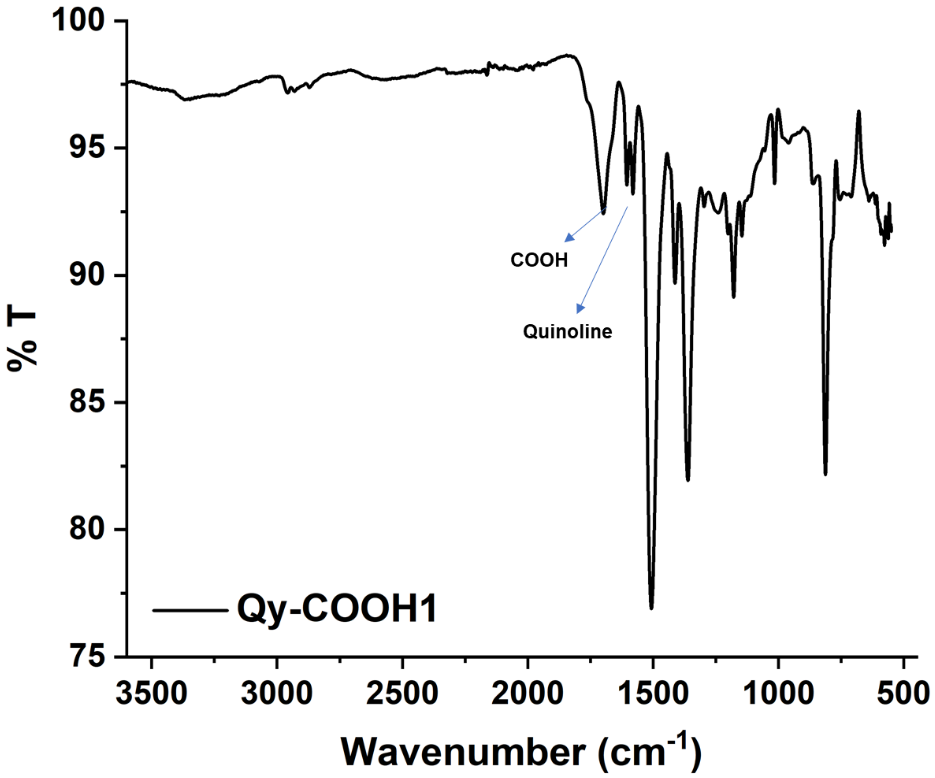

Fig. S21 FTIR spectrum of **Qy-COOH1.**

(a)

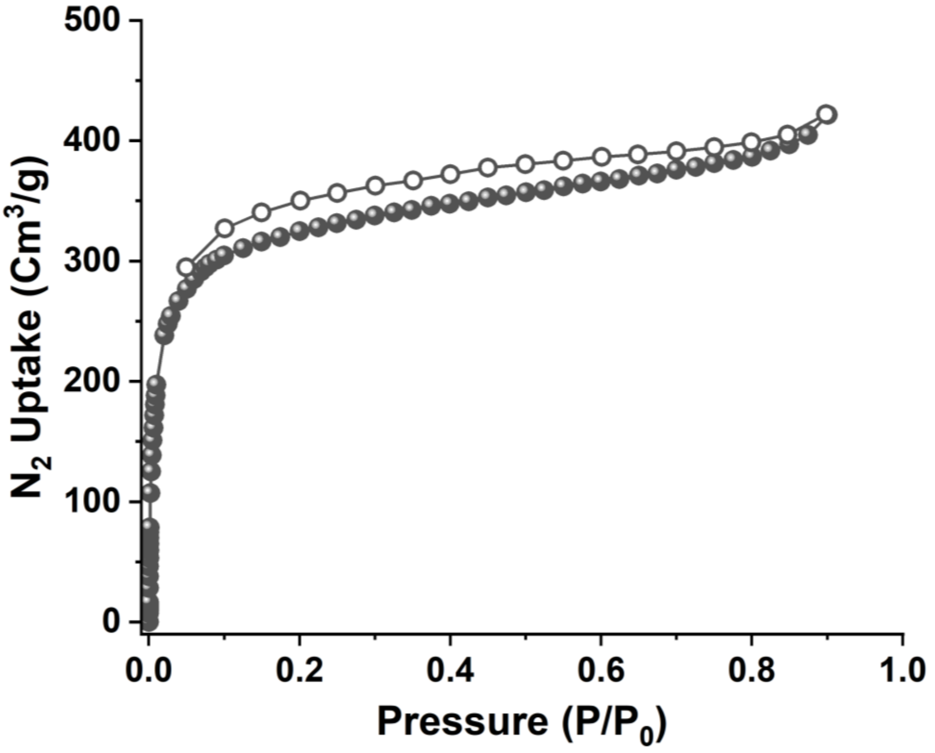

(b)

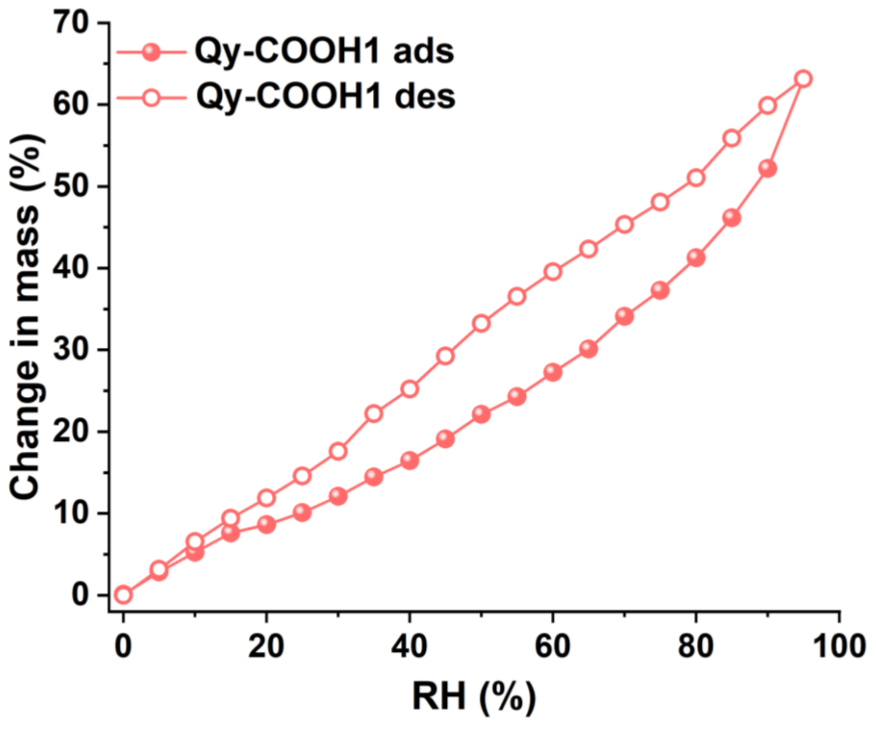

Fig. S22 (a) N_2 sorption isotherm of **Qy-COOH1** at 77 K. (b) Water sorption isotherm at RT.

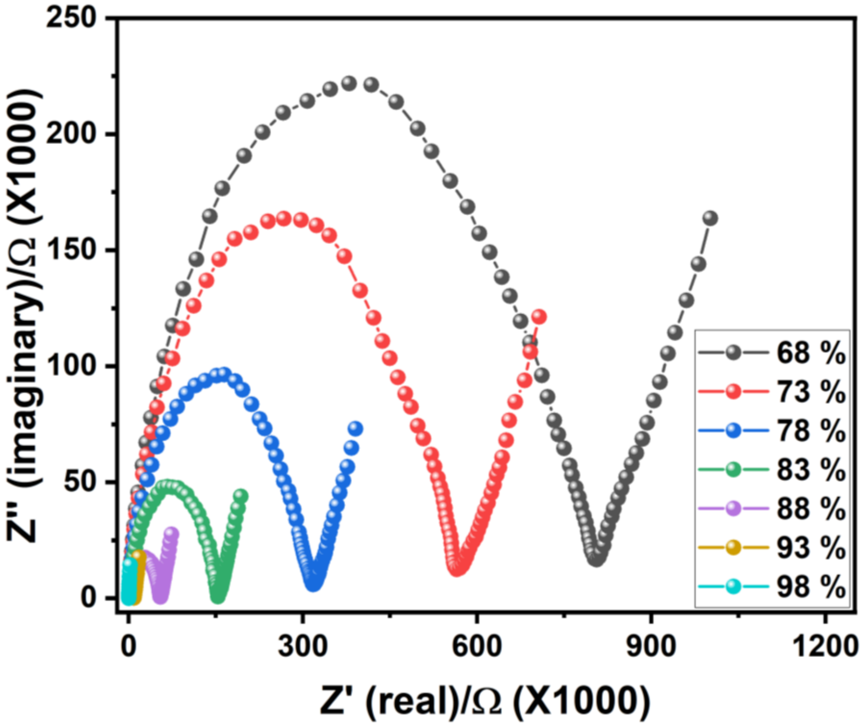

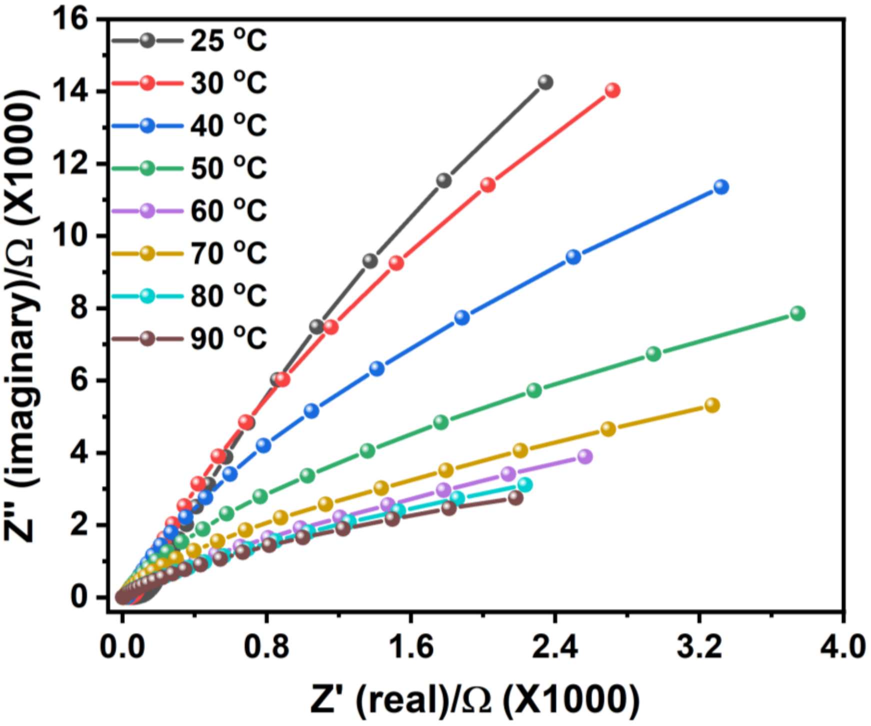

Fig. S23 Nyquist plots of **Qy-COOH1** measured under (top) different humidity at room temperature and (bottom) 98% RH at different temperatures showing the temperature dependence of proton conductivities.

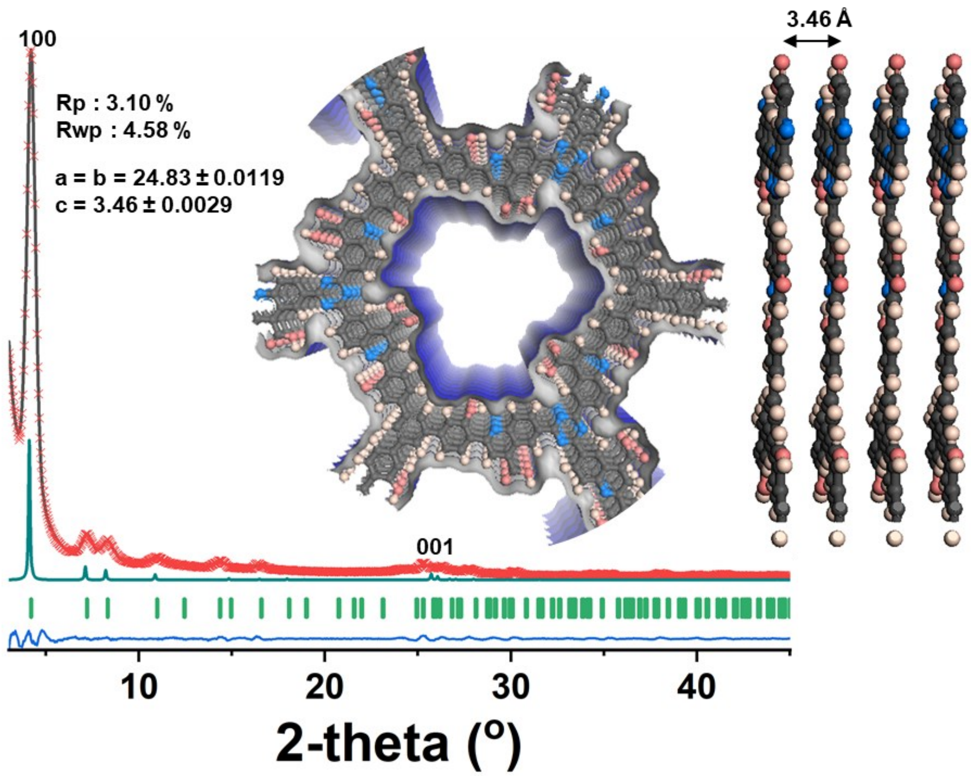

Fig. S24 Simulated and experimental PXRD patterns of **OH-Qy-COOH**. Experimental (black dotted line) and Pawley refined (red cross) patterns, Bragg position (green) and corresponding difference plot (blue line). The theoretical PXRD pattern for an eclipsed AA stacking model of **OH-Qy-COOH** is provided as cyan line. Top and side view of AA stacking in **OH-Qy-COOH**. (Color code: C, gray spheres; O, red spheres; N, blue spheres, H, pale yellow spheres).

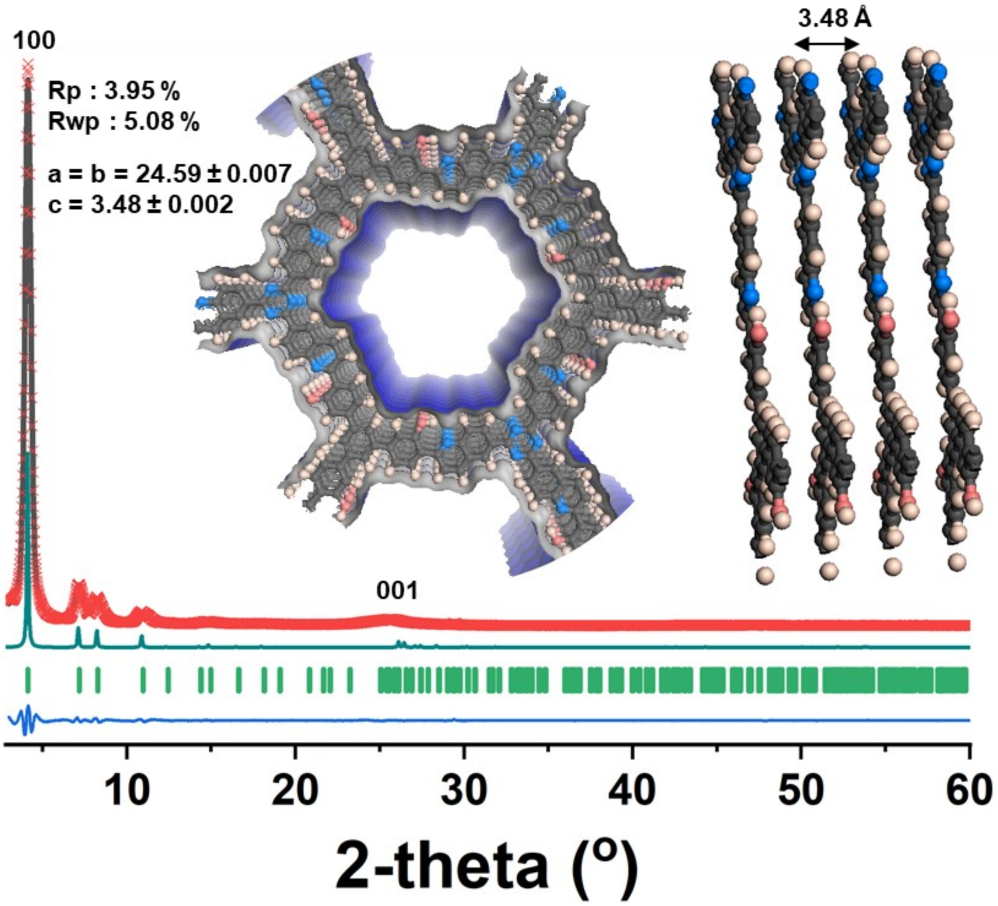

Fig. S25 Simulated and experimental PXRD patterns of **OH-Qy-H**. Experimental (black dotted line) and Pawley refined (red cross) patterns, Bragg position (green) and corresponding difference plot (blue line). The theoretical PXRD pattern for an eclipsed AA stacking model of **OH-Qy-H** is provided as cyan line. Top and side view of AA stacking in **OH-Qy-H**. (Color code: C, gray spheres; O, red spheres; N, blue spheres, H, pale yellow spheres).

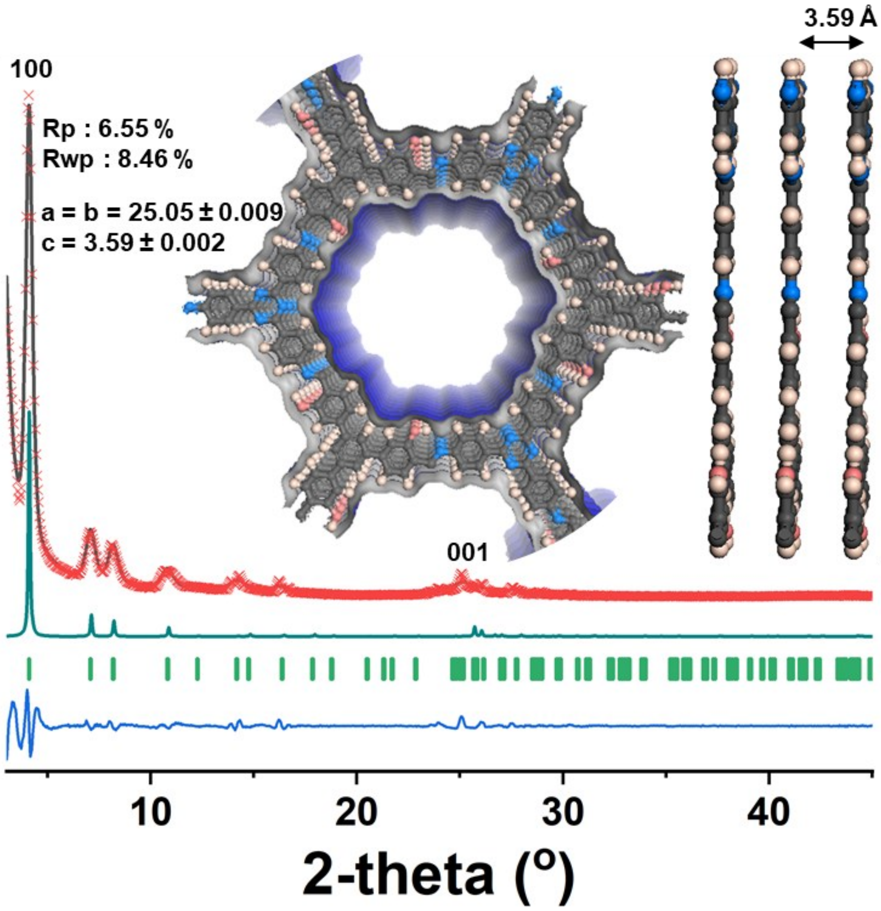

Fig. S26 Simulated and experimental PXRD patterns of **OH-Im**. Experimental (black dotted line) and Pawley refined (red cross) patterns, Bragg position (green) and corresponding difference plot (blue line). The theoretical PXRD pattern for an eclipsed AA stacking model of **OH-Im** is provided as cyan line. Top and side view of AA stacking in **OH-Im**. (Color code: C, gray spheres; O, red spheres; N, blue spheres, H, pale yellow spheres).

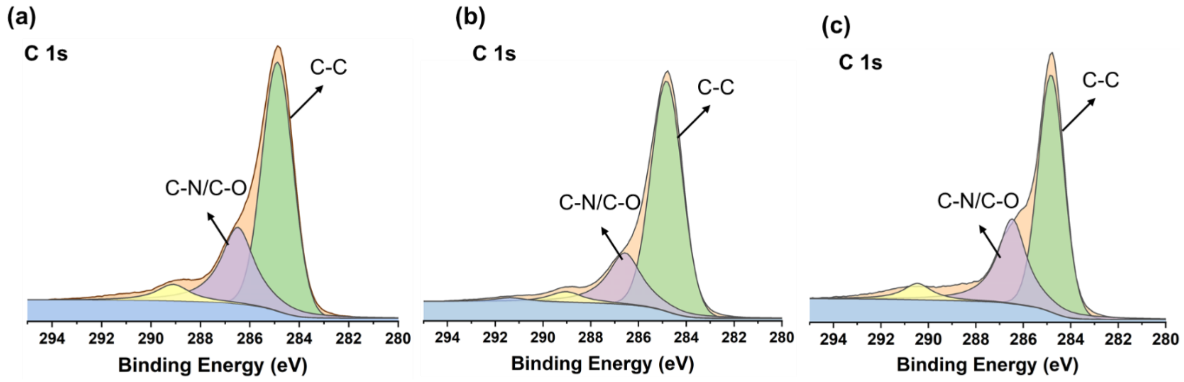

Fig. S27 C(1s) XPS spectra of (a) **OH-Qy-COOH**, (b) **OH-Qy-H** and (c) **OH-Im**.

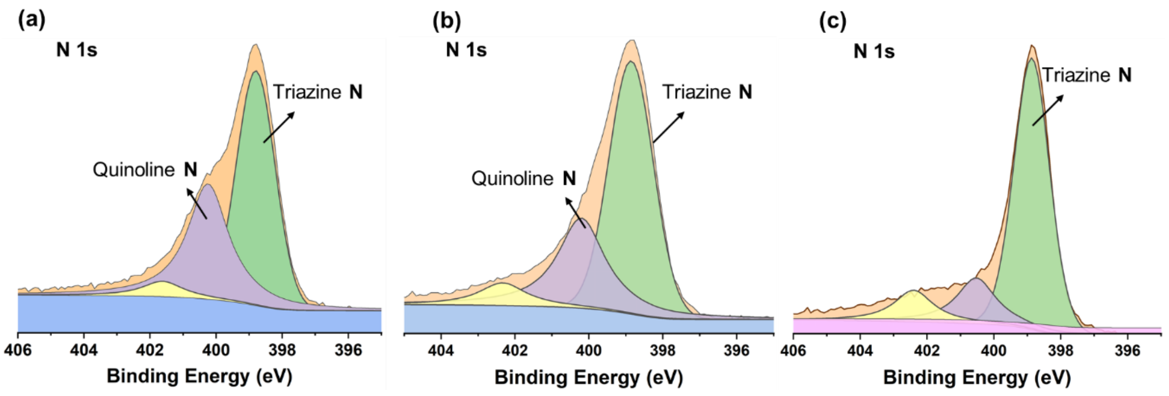

Fig. S28 N(1s) XPS spectra of (a) **OH-Qy-COOH**, (b) **OH-Qy-H** and (c) **OH-Im.**

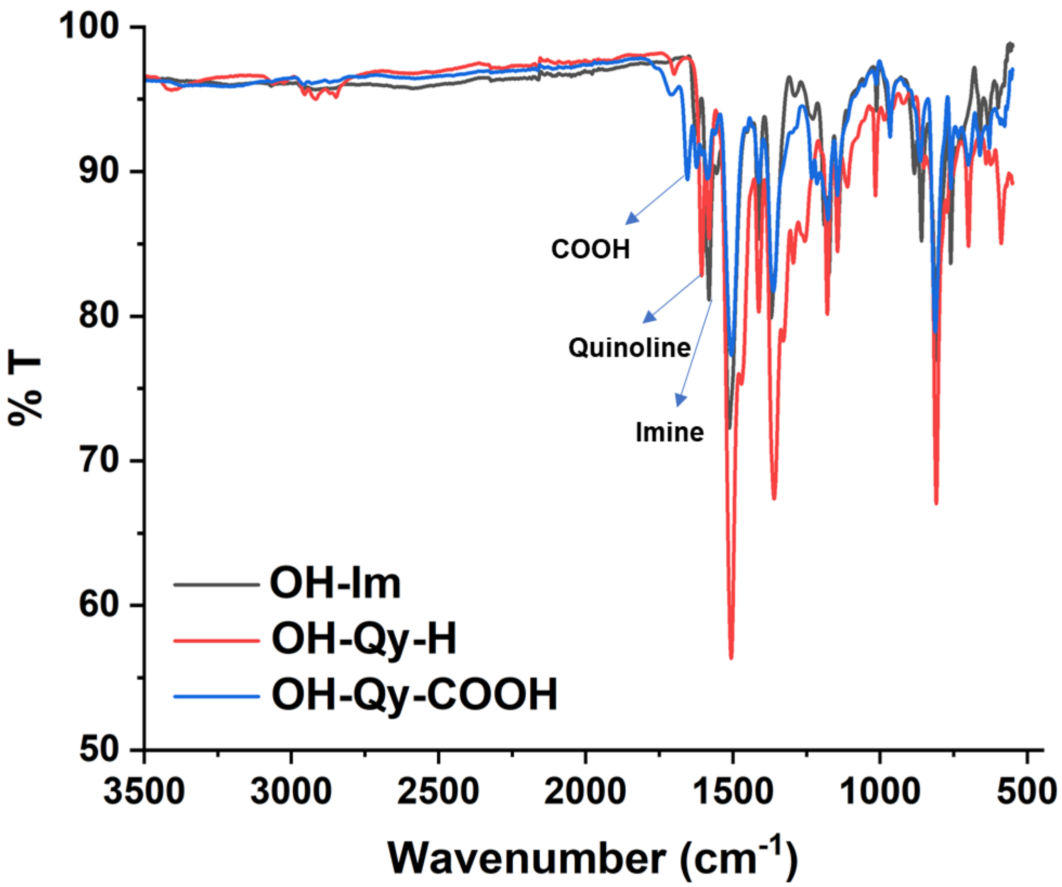

Fig. S29 FTIR spectra of **OH-Qy-COOH, OH-Qy-H and OH-Im.**

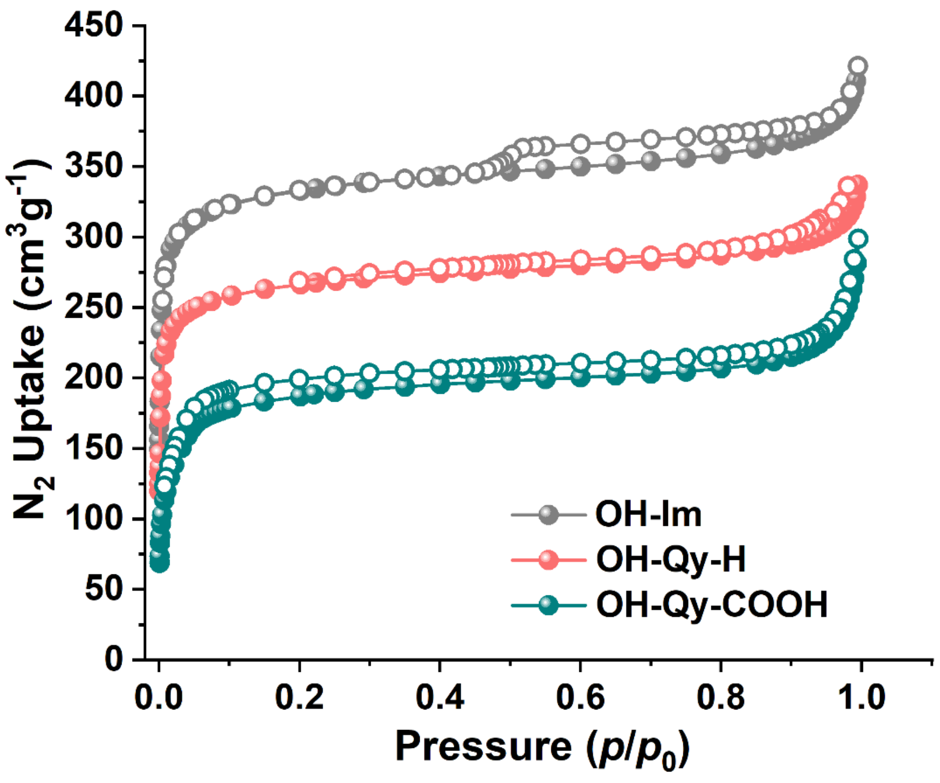

Fig. S30 N_2 sorption isotherms of **OH-Qy-COOH, OH-Qy-H and OH-Im** collected at 77 K. The surface area was found to be 656, 892, 1407 m^2/g for **OH-Qy-COOH, OH-Qy-H and OH-Im**, respectively.

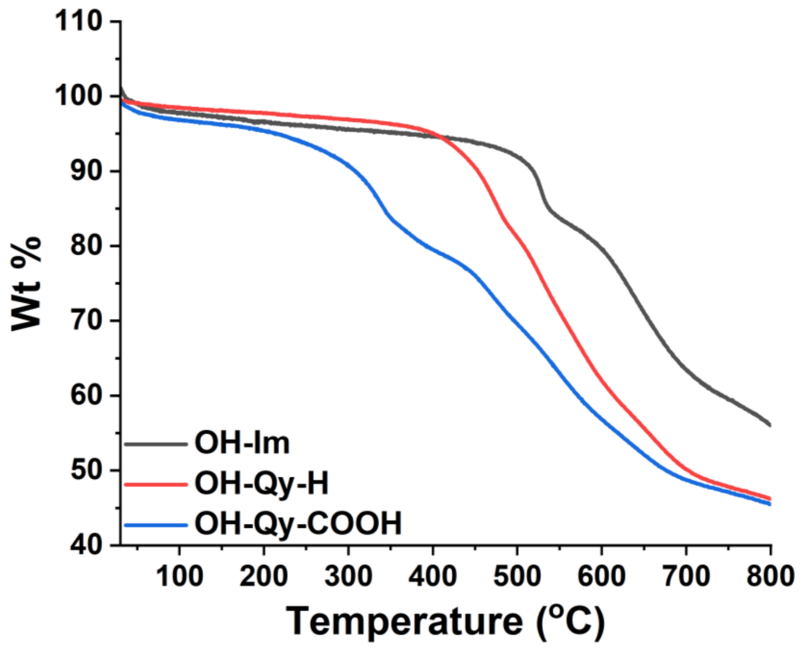

Fig. S31 TGA profiles of **OH-Qy-COOH, OH-Qy-H** and **OH-Im** performed at N_2 atmosphere.

(a)

(b)

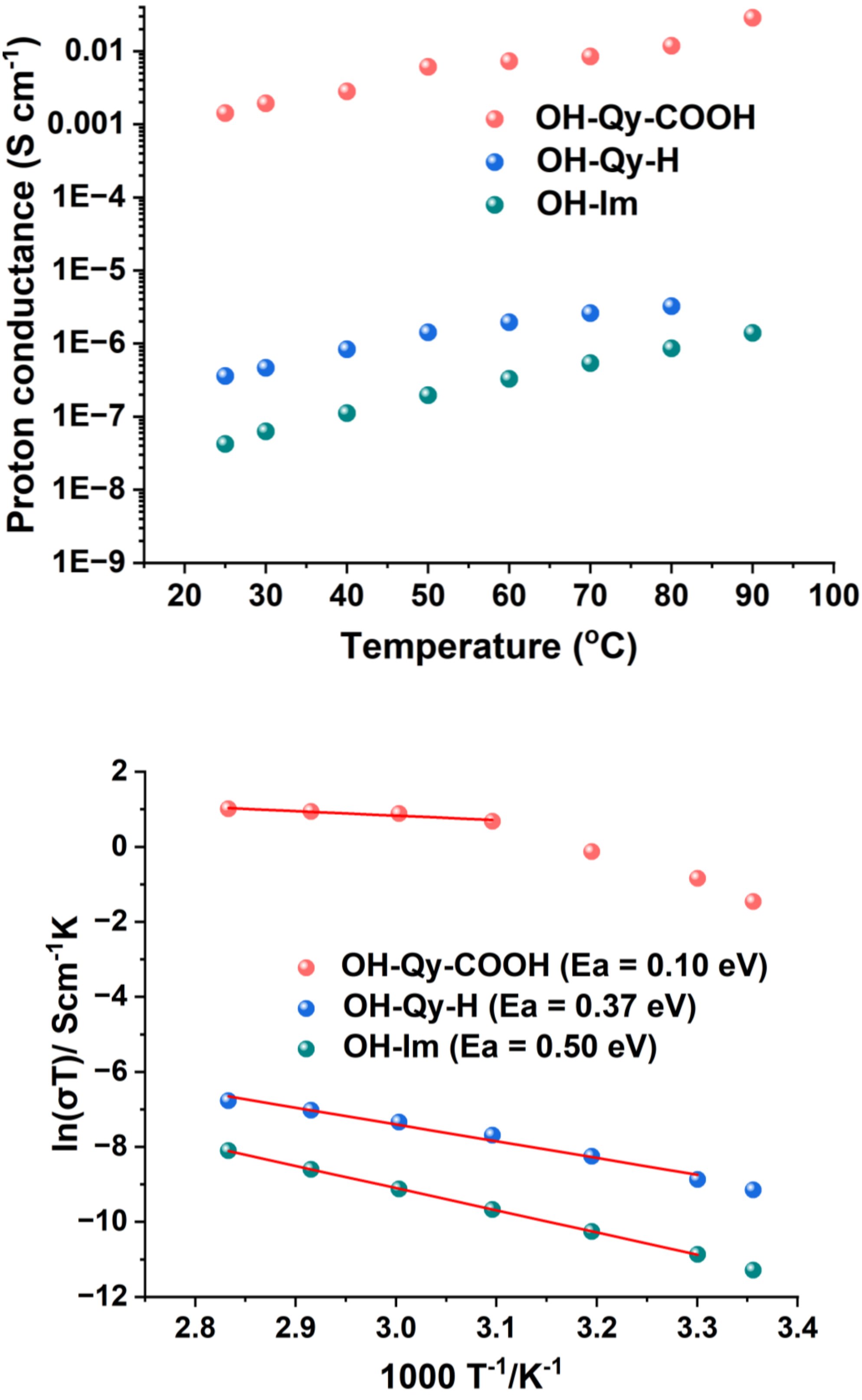

Fig. S32 (a) Proton conductance of **OH-Qy-COOH, OH-Qy-H** and **OH-Im** at different temperature with 98% RH. (b) Arrhenius plots for **OH-Qy-COOH, OH-Qy-H** and **OH-Im** at different temperatures. (Dots for data and curves for fitting).

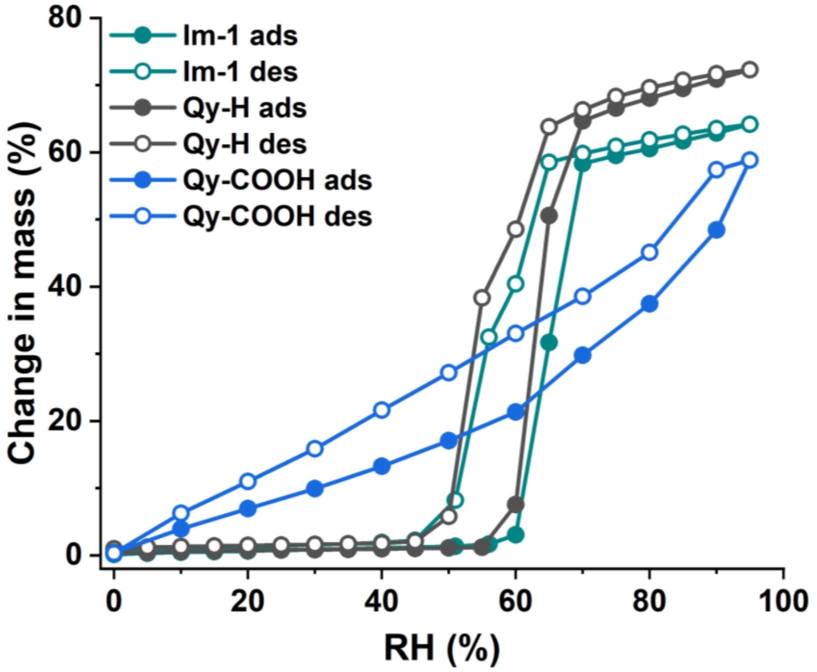

Fig. S33 Water sorption isotherms of **OH-Qy-COOH, OH-Qy-H** and **OH-Im**

(a)

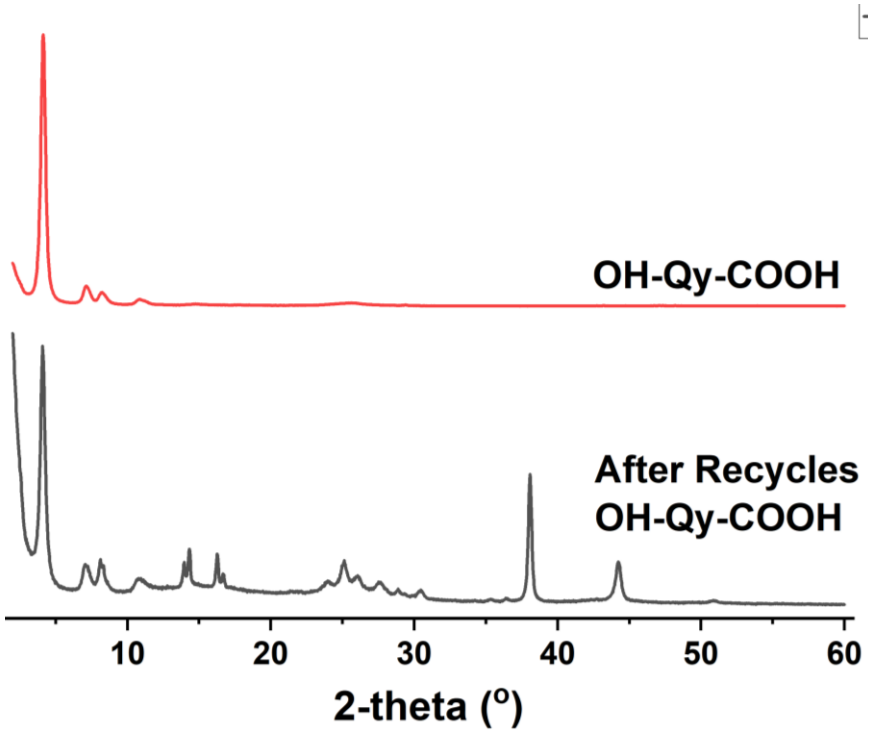

(b)

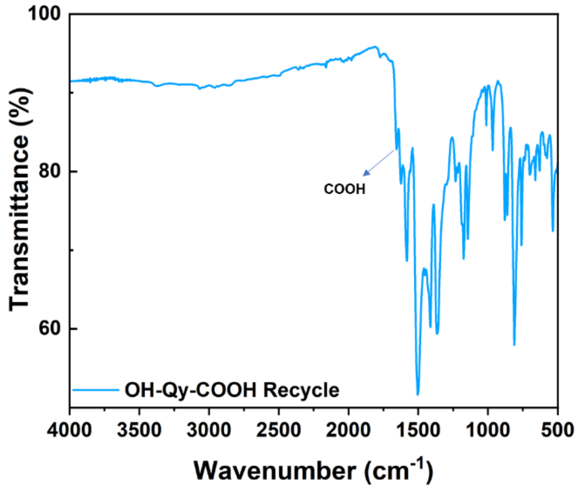

(c)

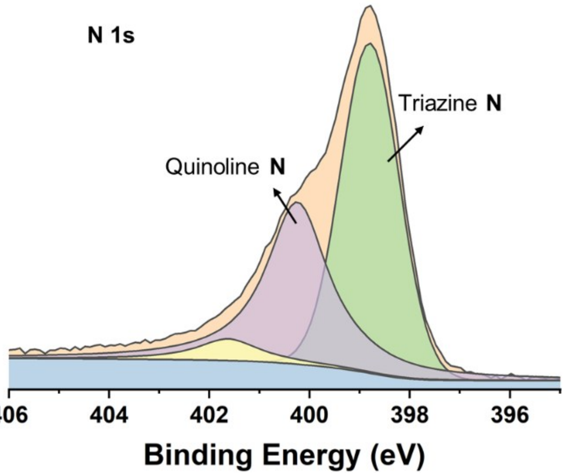

(d)

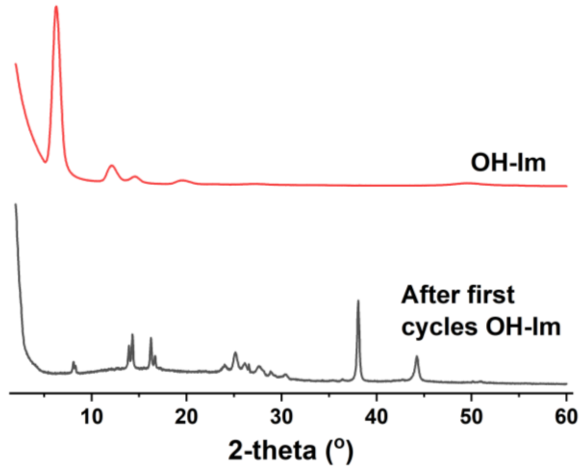

Fig. S34 (a) PXRD patterns (Extra peaks in PXRD pattens correspond to silver coating), (b) FTIR spectrum and (c) N1S XPS spectra of **OH-Qy-COOH** after long-term experiment. (d) PXRD patterns **OH-Im** showing instability after first cycle. (Extra peaks in PXRD pattens correspond to silver coating).

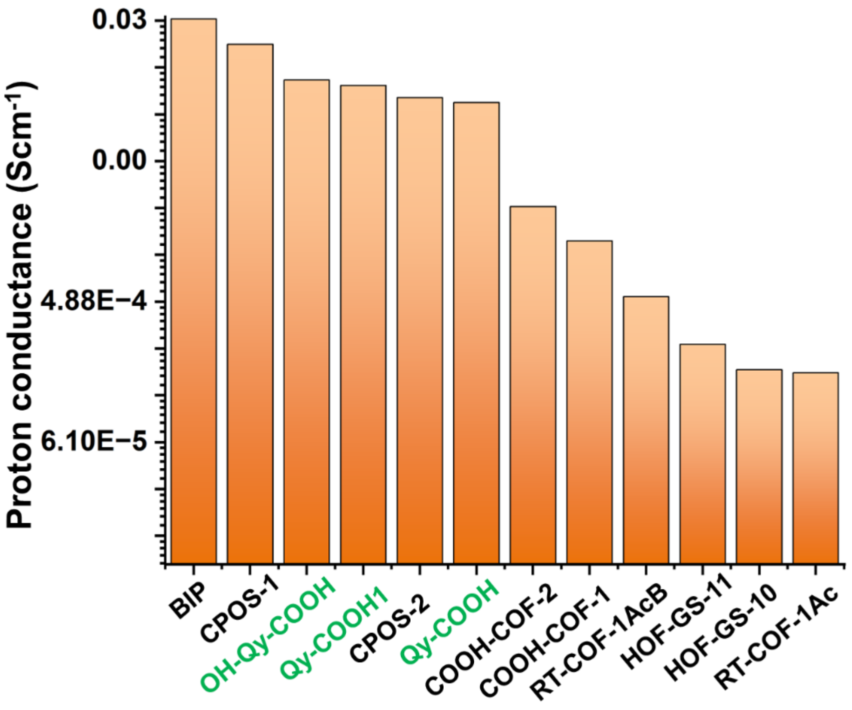

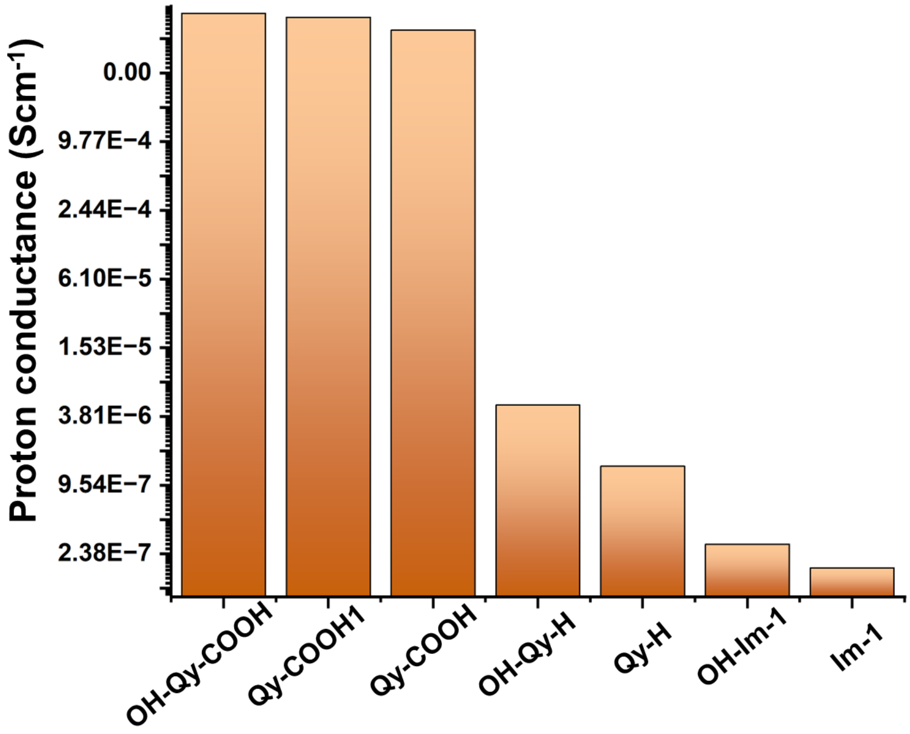

Fig. S35 (Top) Comparing the intrinsic proton conductivities of OH-Qy-COOH, Qy-COOH1, and Qy-COOH with benchmark COFs, porous organic polymers (POP) and hydrogen bonded organic frameworks (HOF)-based proton conductors under humid conditions. (Bottom) Comparing the proton conductivities of OH-Qy-COOH, Qy-COOH1, and Qy-COOH with their corresponding quinoline and imine linked COF.

Table S1. Comparison of Proton Conductance of different materials.

Samples	Proton Conductance (Scm^{-1})	Temperature (°C)	RH (%)	Dopant	Reference
**Qy-COOH**	1.2×10^{-3} / 9.3×10^{-3}	RT / 90	98	Without additives	**This work**
**Qy-COOH1**	1.3×10^{-3} / 1.2×10^{-2}	RT / 90	98	Without additives	
**OH-Qy-COOH**	1.1×10^{-3} / 1.3×10^{-2}	RT / 90	98	Without additives	
im@TPB-DMTP-COF	4.37×10^{-3}	130	0	Imidazole	*Nat. Mater.* **2016**, *15*, 722.
trz@TPB-DMTP-COF	1.10×10^{-3}	130	0	triazole	
HOF-GS-10	1.78×10^{-4}	30	60	-	*Angew. Chem. Int. Ed.* **2016**, *55*, 10667.
HOF-GS-11	2.60×10^{-4}	30	60	-	
RT-COF-1AcB	5.25×10^{-4}	40	100	-	*J. Am. Chem. Soc.* **2017**, *139*, 10079.
RT-COF-1Ac	1.07×10^{-4}	40	100	-	
LiCl@RT-COF-1	6.45×10^{-3}	40	100	LiCl	
PA@TpBpy-MC	2.50×10^{-3}	120	0	H_3PO_4	*J. Mater. Chem. A* ***2016**, 4,* 2682.
PA@TpBpy-ST	1.98×10^{-3}	120	0	H_3PO_4	
phytic@TpPa-Py	3.00×10^{-4}	120	0	Phytic acid	*Chem. Mater.* ***2016,** 28,* 1489.
PA@Tp-Azo	9.90×10^{-4}	60	98	H_3PO_4	*J. Am. Chem. Soc.* **2014***, 136,* 6570.
PA@Tp-Stb	2.30×10^{-5}	60	98	H_3PO_4	
H_3PO_4@NKCOF-1	1.13×10^{-1}	80	98	H_3PO_4	*Angew. Chem. Int. Ed.* **2020**, *59*, 3678.
H_3PO_4@NKCOF-2	4.28×10^{-2}	80	98	H_3PO_4	
H_3PO_4@NKCOF-3	1.12×10^{-2}	80	98	H_3PO_4	
H_3PO_4@NKCOF-4	7.71×10^{-2}	80	98	H_3PO_4	
H_3PO_4@TPB-DMeTP-COF	1.91×10^{-1}	160	0	H_3PO_4	*Nat. Commun.* **2020**, *11*, 1981
BIP-COF	3.20×10^{-2}	95	95	-	*J. Am. Chem. Soc.* **2019**, *141*, 14950
COOH-COF-1	1.23×10^{-3}	80	98	-	*Chinese Chemical Letters* **2023**, *34*, 107917
COOH-COF-2	2.4×10^{-3}	80	98	-	

Aza-COF-1	1.23 × 10^{-3}	50	97	H_3PO_4	*Chem. Mater.* **2019**, *31*, 819.
Aza-COF-2	4.80 × 10^{-3}	50	97	H_3PO_4	
PTSA@TpAzo COFM	7.80×10^{-2}	80	95	PTSA	*Angew. Chem. Int. Ed.* **2018**, *57*, 10894.
PTSA@TpBpy COFM	6.20×10^{-2}	80	95	PTSA	
PTSA@TpBD(Me)2 COFM	5.3×10^{-2}	80	95	PTSA	
CPOS-1	1.00×10^{-2}	60	98	-	*Angew. Chem. Int. Ed.* **2018**, *57*, 1.
CPOS-2	2.2×10^{-2}	60	98	-	
PA@NKCOF-54	2.33×10^{-2}	160	0	H_3PO_4	*Angew Chem Int Ed.* **2023**, *62, e202217240.*
PIL@m-TpPa-SO_3H	1.02×10^{-1}	90	100	Ionic Liquid	*Adv. Funct. Mater.* **2023**, 33.
PA@PyPz-COF	8.10×10^{-2}	80	98	H_3PO_4	*Small* **2023**, *19,* e2207421.
PA@PBI-COF	1.57×10^{-1}	160	0	H_3PO_4	*ACS Mater. Letters* **2022**, *4,* 2597-2603.
PA@TPB-DABI-COF	1.91×10^{-1}	160	0	H_3PO_4	*Angew Chem Int Ed.* **2021**, *60,* 12918-12923.
COF-F6-H	4.2×10^{-2}	140	0	-	*J. Am. Chem. Soc.* **2020**, *142,* 14357–14364.
PA@CTF-TF	1.82×10^{-1}	150	0	H_3PO_4	*Nat. Commun.* **2023**, *14,* 8114.
PA@MTI-DAPy-COF	3.68×10^{-2}	150	0	H_3PO_4	*Sci. Rep.* **2025**, *15,* 5758.
Aza-TT	3.4 × 10^{-2}	95	98	TFA	*J. Am. Chem. Soc.* **2024**, *146,* 23497–23507

Table S2. Fractional coordinates for Qy-COOH

Qy-COOH_Pawley				
Space Group: $P\bar{6}$ (174)<br>*a* = 25.9289 Å, *b* = 25.9289 Å, *c* = 3.4368 Å<br>$\alpha = \beta = 90.00°$, $\gamma = 120.00°$				
**Atom label**	**Atom type**	***x***	***y***	***z***
C1	C	0.61275	0.71061	0.00000
C2	C	0.59951	0.75837	0.00000
C3	C	0.64387	0.81902	0.00000
C4	C	0.70396	0.83591	0.00000
C5	C	0.71999	0.79209	0.00000
C6	C	0.67609	0.73138	0.00000
H7	H	0.55722	0.75472	0.00000
H8	H	0.63126	0.85338	0.00000
H9	H	0.73806	0.88291	0.00000
H10	H	0.76705	0.80513	0.00000
H11	H	0.69587	0.70346	0.00000
C12	C	0.58967	0.60193	0.00000
C13	C	0.56564	0.64154	0.00000
H14	H	0.63613	0.61814	0.00000
C15	C	0.27216	0.63441	0.00000
N16	N	0.30469	0.60604	0.00000
C17	C	-0.20765	-0.62328	0.00000
C18	C	-0.18558	-0.66330	0.00000
C19	C	-0.12388	-0.64215	0.00000
C20	C	-0.08208	-0.57992	0.00000
C21	C	-0.10491	-0.54018	0.00000
C22	C	-0.16657	-0.56146	0.00000
N23	N	0.02005	-0.49452	0.00000
C24	C	-0.01524	-0.55449	0.00000
C25	C	0.11130	-0.49984	0.00000
C26	C	0.08034	-0.46657	0.00000
C27	C	0.11065	-0.40389	0.00000
C28	C	0.17260	-0.37110	0.00000
C29	C	0.20550	-0.40068	0.00000
C30	C	0.17484	-0.46392	0.00000
H31	H	-0.21603	-0.71132	0.00000
H32	H	-0.11058	-0.67572	0.00000
H33	H	-0.07514	-0.49206	0.00000
H34	H	-0.18193	-0.52923	0.00000
H35	H	0.08566	-0.38026	0.00000
H36	H	0.19458	-0.32257	0.00000
H37	H	0.20361	-0.48110	0.00000
N39	N	0.60690	0.31270	0.00000

Table S3. Fractional coordinates for Qy-H

**Qy-H_Pawley**				
Space Group: $P\bar{6}$ (174)<br>a = 24.8447 Å, b = 24.8447 Å, c = 3.4103 Å<br>$\alpha = \beta$ = 90.00°, γ = 120.00°				
**Atom label**	**Atom type**	***x***	***y***	***z***
C1	C	0.27049	0.63666	-1
N2	C	0.30104	0.60487	-1
C3	C	-0.20782	-0.61808	-1
C4	C	-0.18321	-0.65549	-1
C5	C	-0.12158	-0.63171	-1
C6	C	-0.08273	-0.56968	-1
C7	C	-0.10774	-0.53243	-1
C8	C	-0.16934	-0.55625	-1
N9	N	0.01824	-0.48248	-1
C10	C	-0.01671	-0.54275	-1
C11	C	0.10831	-0.48744	-1
C12	C	0.07863	-0.45441	-1
C13	C	0.11149	-0.39236	-1
C14	C	0.17358	-0.36285	-1
C15	C	0.20418	-0.39498	-1
C16	C	0.17068	-0.45769	-1
H17	H	-0.21155	-0.70339	-1
H18	H	-0.10538	-0.66269	-1
H19	H	-0.07976	-0.4845	-1
H20	H	-0.1867	-0.52603	-1
H21	H	0.08895	-0.36676	-1
H22	H	0.19748	-0.31471	-1
H23	H	0.19289	-0.48364	-1
C24	C	0.64339	0.3702	0
N25	N	0.60698	0.31023	0
C26	C	0.57874	0.59202	1
C27	C	0.54963	0.62478	1
C28	H	0.57574	0.67292	1
H32	H	0.62671	0.61582	1

Table S4. Fractional coordinates for Qy-COOH1

Qy-COOH1_Pawley				
Space Group: $P\bar{6}$ (174)<br>a = 23.9440 Å, b = 23.9440 Å, c = 3.4277 Å<br>$\alpha = \beta$ = 90.00°, γ = 120.00°				
**Atom label**	**Atom type**	***x***	***y***	***z***
N1	N	0.66667	0.33333	-1
C2	C	0.26758	0.63386	-1
N3	N	0.30066	0.60145	-1
C4	C	-0.25855	-0.6403	-1
C5	C	-0.22659	-0.67802	-1
C6	C	-0.15983	-0.6553	-1
C7	C	-0.11543	-0.59031	-1
C8	C	-0.14062	-0.54969	-1
C9	C	-0.20753	-0.5735	-1
N10	N	-0.00469	-0.49879	-1
C11	C	-0.04399	-0.56363	-1
C12	C	0.09322	-0.50452	-1
C13	C	0.06054	-0.46885	-1
C14	C	0.09505	-0.40151	-1
C15	C	0.16209	-0.36817	-1
C16	C	0.19604	-0.40179	-1
C17	C	0.16137	-0.46967	-1
H18	H	-0.24311	-0.7258	-1
H19	H	-0.14366	-0.69018	-1
H20	H	-0.10772	-0.49784	-1
H21	H	-0.20691	-0.53099	-1
H22	H	0.06967	-0.37466	-1
H23	H	0.18725	-0.31597	-1
H24	H	0.18896	-0.49356	0
C25	C	0.60473	0.59071	0
C26	C	0.57715	0.6305	1
C27	C	0.62231	0.70201	1
O28	O	0.60029	0.74591	1
O29	O	0.68047	0.72301	1
H30	H	0.63652	0.79246	1
H31	H	0.65645	0.6126	1

Table S5. Fractional coordinates for OH-Qy-H

OH-Qy-H_Pawley				
Space Group: $P\bar{6}$ (174)<br>a = 24.5966 Å, b = 24.5966 Å, c = 3.4862 Å<br>$\alpha = \beta$ = 90.00°, γ = 120.00°				
**Atom label**	**Atom type**	***x***	***y***	***z***
H3	H	0.59311	0.68275	0.00000
H1	H	0.44542	0.41005	0.00000
H2	H	0.73485	0.44132	0.00000
C29	C	0.58652	0.60003	0.00000
C30	C	0.55905	0.63612	0.00000
H31	H	0.63432	0.61986	0.00000
C4	C	0.27186	0.63518	0.00000
N5	N	0.30362	0.60585	0.00000
C6	C	-0.20873	-0.62146	0.00000
C7	C	-0.18442	-0.65979	0.00000
C8	C	-0.12203	-0.6361	0.00000
C9	C	-0.08167	-0.57262	0.00000
C10	C	-0.10788	-0.5339	0.00000
C11	C	-0.16957	-0.55945	0.00000
N12	N	0.02225	-0.48971	0.00000
C13	C	-0.01513	-0.54914	0.00000
C14	C	0.11292	-0.49442	0.00000
C15	C	0.08255	-0.46139	0.00000
C16	C	0.1135	-0.39865	0.00000
C17	C	0.17585	-0.36664	0.00000
C18	C	0.20856	-0.39721	0.00000
C19	C	0.17674	-0.46087	0.00000
H20	H	-0.21329	-0.70839	0.00000
H21	H	-0.10723	-0.66892	0.00000
O22	O	-0.08048	-0.47046	0.00000
H23	H	-0.18615	-0.52791	0.00000
H24	H	0.08907	-0.37431	0.00000
H25	H	0.19794	-0.31779	0.00000
H26	H	0.20296	-0.48257	0.00000
C27	C	0.64375	0.37269	0.00000
C28	C	0.60527	0.31233	0.00000

Table S6. Fractional coordinates for OH-Qy-COOH

**OH-Qy-COOH_Pawley**				
Space Group: $P\bar{6}$ (174)<br>*a* = 24.8320 Å, *b* = 24.8320 Å, *c* = 3.4613 Å<br>$\alpha = \beta = 90.00°$, $\gamma = 120.00°$				
**Atom label**	**Atom type**	***x***	***y***	***z***
H1	H	0.44542	0.41005	-1
H2	H	0.73485	0.44132	-1
C28	C	0.58652	0.60003	-1
C29	C	0.55905	0.63612	-1
C30	C	0.60053	0.70193	-1
O31	O	0.5794	0.74214	-1
O32	O	0.65478	0.7215	-1
H33	H	0.61247	0.78538	-1
H34	H	0.63432	0.61986	-1
C3	C	0.27186	0.63518	-1
N4	N	0.30362	0.60585	-1
C5	C	-0.20873	-0.62146	-1
C6	C	-0.18442	-0.65979	-1
C7	C	-0.12203	-0.6361	-1
C8	C	-0.08167	-0.57262	-1
C9	C	-0.10788	-0.5339	-1
C10	C	-0.16957	-0.55945	-1
N11	N	0.02225	-0.48971	-1
C12	C	-0.01513	-0.54914	-1
C13	C	0.11292	-0.49442	-1
C14	C	0.08255	-0.46139	-1
C15	C	0.1135	-0.39865	-1
C16	C	0.17585	-0.36664	-1
C17	C	0.20856	-0.39721	0
C18	C	0.17674	-0.46087	0
H19	H	-0.21329	-0.70839	1
H20	H	-0.10723	-0.66892	1
O21	O	-0.08048	-0.47046	1
H22	H	-0.18615	-0.52791	1
H23	H	0.08907	-0.37431	-1
H24	H	0.19794	-0.31779	-1
H25	H	0.20296	-0.48257	-1
C26	C	0.64375	0.37269	0
C27	C	0.60527	0.31233	0

Table S7. Fractional coordinates for OH-Im

**OH-Im_Pawley**				
Space Group: $P\bar{6}$ (174)<br>a = 25.0533 Å, b = 25.0533 Å, c = 3.5948 Å<br>$\alpha = \beta$ = 90.00°, γ = 120.00°				
**Atom label**	**Atom type**	***x***	***y***	***z***
H1	C	0.27049	0.63666	-1
H2	C	0.30104	0.60487	-1
H28	C	-0.20782	-0.61808	-1
C3	C	-0.18321	-0.65549	-1
N4	C	-0.12158	-0.63171	-1
C5	C	-0.08273	-0.56968	-1
C6	C	-0.10774	-0.53243	-1
C7	C	-0.16934	-0.55625	-1
C8	N	0.01824	-0.48248	-1
C9	C	-0.01671	-0.54275	-1
C10	C	0.10831	-0.48744	-1
N11	C	0.07863	-0.45441	-1
C12	C	0.11149	-0.39236	-1
C13	C	0.17358	-0.36285	-1
C14	C	0.20418	-0.39498	-1
C15	C	0.17068	-0.45769	-1
C16	H	-0.21155	-0.70339	-1
C17	H	-0.10538	-0.66269	-1
C18	H	-0.07976	-0.4845	-1
H19	H	-0.1867	-0.52603	-1
H20	H	0.08895	-0.36676	-1
O21	H	0.19748	-0.31471	-1
H22	H	0.19289	-0.48364	-1
H23	C	0.64339	0.3702	0
H24	N	0.60698	0.31023	0
H25	C	0.57874	0.59202	1
C26	C	0.54963	0.62478	1
C27	H	0.57574	0.67292	1
